# Engineered Branaplam Aptamers Exploit Structural Elements from Natural Riboswitches

**DOI:** 10.1021/acschembio.4c00358

**Published:** 2024-07-02

**Authors:** Michael G. Mohsen, Matthew K. Midy, Aparaajita Balaji, Ronald R. Breaker

**Affiliations:** † Department of Molecular, Cellular and Developmental Biology, Yale University, New Haven, Connecticut 06511, United States; ‡ Department of Molecular Biophysics and Biochemistry, Yale University, New Haven, Connecticut 06511, United States; § Howard Hughes Medical Institute, Yale University, New Haven, Connecticut 06511, United States

## Abstract

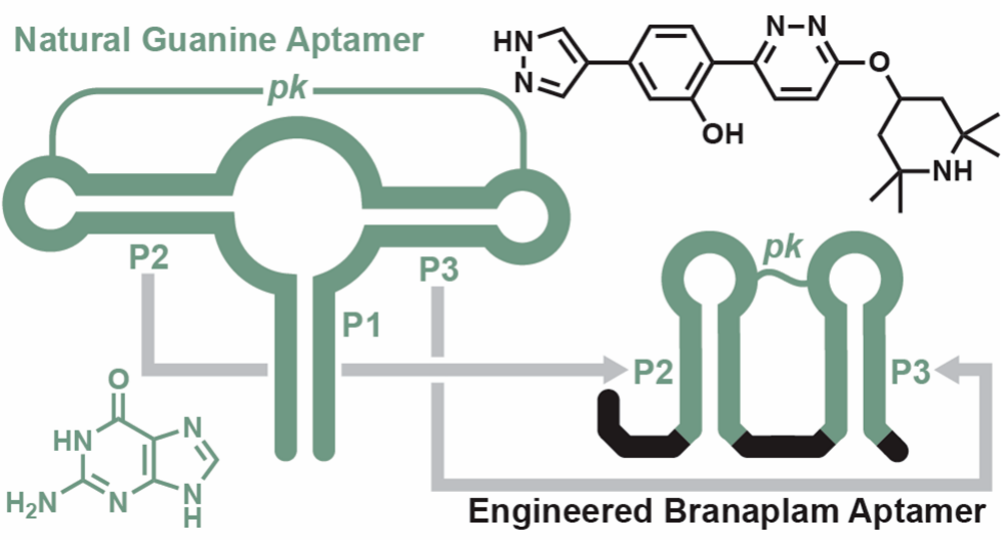

Drug candidates that fail in clinical trials for efficacy reasons might still have favorable safety and bioavailability characteristics that could be exploited. A failed drug candidate could be repurposed if a receptor, such as an aptamer, were created that binds the compound with high specificity. Branaplam is a small molecule that was previously in development to treat spinal muscular atrophy and Huntington’s disease. Here, we report the development of a small (48-nucleotide) RNA aptamer for branaplam with a dissociation constant of ∼150 nM. Starting with a combinatorial RNA pool integrating the secondary and tertiary structural scaffold of a Guanine-I riboswitch aptamer interspersed with regions of random sequence, in vitro selection yielded aptamer candidates for branaplam. Reselection and rational design were employed to improve binding of a representative branaplam aptamer candidate. A resulting variant retains the pseudoknot and two of the paired elements (P2 and P3) from the scaffold but lacks the enclosing paired element (P1) that is essential for the function of the natural Guanine-I riboswitch aptamer. A second combinatorial RNA pool based on the scaffold for TPP (thiamin pyrophosphate) riboswitches also yielded a candidate offering additional opportunities for branaplam aptamer development.

RNA aptamers form binding pockets that recognize a target ligand.^1,2^ There is an increasing interest in aptamers that bind drug or drug-like compounds for implementation in synthetic biology and biomedicine.^3,4^ One intriguing prospect is the application of engineered aptamers as components of gene regulation devices for human gene therapy.^5−7^

Typically, drug candidates are developed to bind a natural biochemical target such as a protein of interest.^8,9^ Successful candidates bind their targets to bring about their desired biological effects, and also possess positive bioavailability and safety characteristics. In some cases, candidates fail to bring the desired biological effects but retain their other positive features. These failed candidates might be made to serve as ligands for engineered aptamers, such that compounds with known safety and bioavailability metrics are repurposed for a different application than that for which they were originally developed.^10^

Natural and engineered RNA aptamers are versatile as receptors, as demonstrated by the vast chemical diversity of previously reported ligands including nucleotides, amino acids, elemental ions, and drugs.^11−14^ This established functional diversity suggests that RNA aptamers could be developed to bind many additional compounds that exhibit attractive drug-like features. These aptamers could be valuable in biomedical applications, such as in regulatory devices for gene therapy constructs. Thus, instead of optimizing ligand candidates for efficacy first, and safety and bioavailability later, only candidates that are highly bioavailable and safe are chosen.

Branaplam, also called LMI070, is an orally available small molecule splicing modulator that was previously under development for treating Type 1 spinal muscular atrophy (SMA) and, subsequently, Huntington’s Disease.^15−19^ Branaplam was reported to have no impact on neurogenesis in juvenile mice, rats, and dogs.^20^ Moreover, according to clinical trial reports related to SMA, branaplam was determined to be safe and well-tolerated in human patients.^21,22^ At the outset of this project, we assessed that branaplam would be a good candidate to be repurposed as a ligand for an engineered RNA aptamer. However, it was recently reported that branaplam induces peripheral neuropathy, which led to the suspension of clinical trials related to Huntington’s Disease.^23^ Thus, a successful repurposing of branaplam as an aptamer ligand would require doses below the level of neurological toxicity.

Previous engineering efforts that exploited structural components of naturally occurring riboswitch aptamers have yielded aptamers for different compounds that function in cells.^24−28^ Thus, we sought to develop an aptamer for branaplam following our previously reported protocol^24,25^ for performing in vitro selection featuring a combinatorial RNA pool that contains structural elements from a Guanine-I riboswitch aptamer^26^ (Figure 1A). This general scaffold is interspersed with regions of random sequence that replace the original ligand-binding pocket.^24−27^ For this effort, we prepared six scaffolded designs (S7 through S12), each containing random regions of differing lengths between the base-paired elements P1, P2, and P3, which are kept constant between the designs (Figure 1B). This approach maintains the predominant structural elements from the natural Guanine-I riboswitch, including a short pseudoknot between the loops of P2 and P3 (L2 and L3). In addition, the shuffling of random-sequence regions between the six designs increases conformational diversity among members of the combinatorial pool.

**Figure 1:**
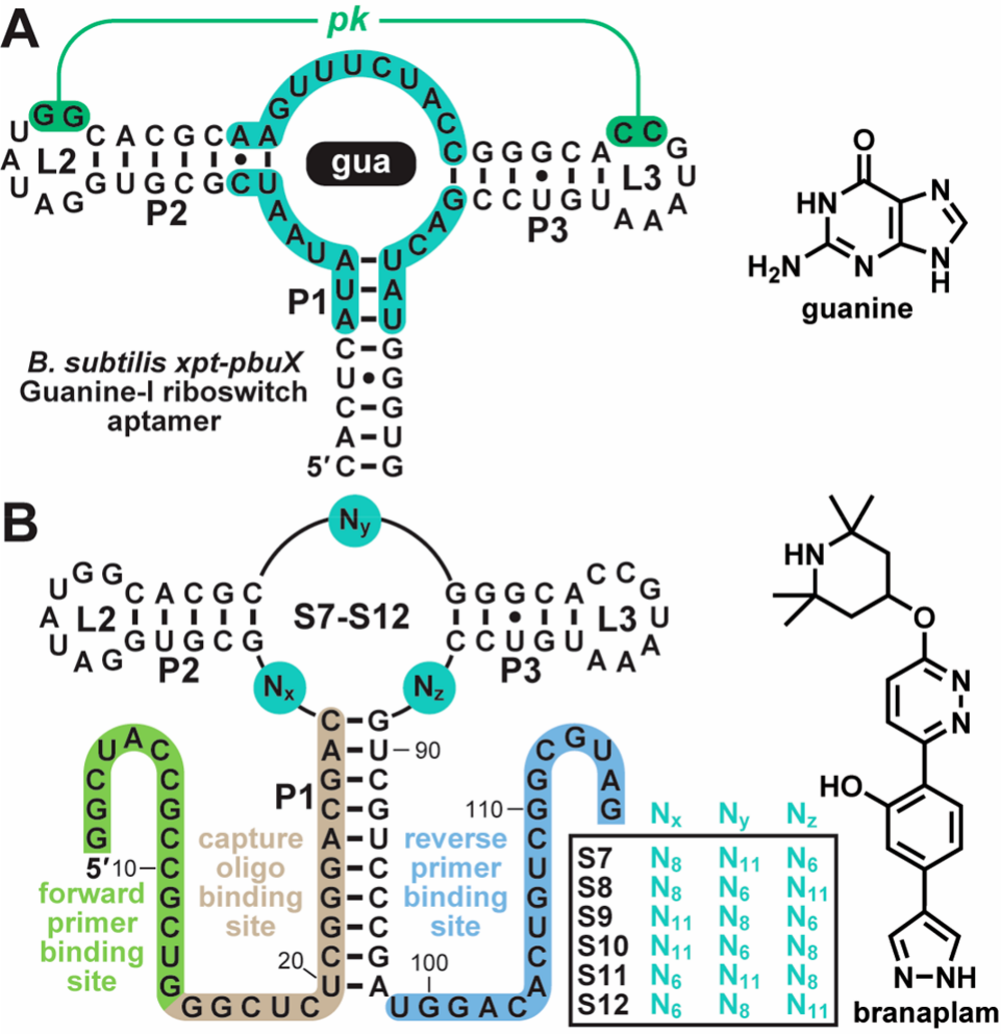
Scaffolds and compounds employed in this study. A. Sequence and secondary structure model of the Guanine-I riboswitch aptamer found upstream of *xpt-pbuX* genes in *Bacillus subtilis*.^26^ Three paired elements (P1–P3) form a three-stem junction, which contains the binding pocket for the target ligand, guanine (right). The two loop regions, designated L2 and L3, form a pseudoknot (pk).^36^ B. Scaffolds S7–S12 contain the sequences of P1–P3, L2, and L3 from the Guanine-I riboswitch aptamer depicted in A with interspersed regions of random sequence N_*x*_, N_*y*_, and N_*z*_ which vary in length. Branaplam (right) is the target ligand for this selection.

In a recent report, a modified DNA synthesis strategy was used to promote stochastic nucleotide insertions and deletions, thereby achieving even greater conformational diversity.^28^ We chose not to use this strategy, so that the lengths of all RNA molecules in the pool are approximately equivalent. This permits simultaneous copurification of representatives from all six scaffolds based on length by polyacrylamide gel electrophoresis (PAGE).

In the six scaffolds, P1 overlaps with the binding site of a 3′-biotinylated capture oligonucleotide (Figure 1B). This design enabled us to perform in vitro selection using the Capture-SELEX^29−32^ strategy. After 11 rounds of in vitro selection, the resulting generation 11 (G11) RNA population was sequenced by using next-generation sequencing (NGS), yielding sequences for ∼40 million members of the population. The most abundant sequence, named 11–1, constitutes 13.1% of the population but many other diverse representatives also exist. Based on the spacing between constant regions, we determined that 11–1 likely originates from scaffold S8 (Figure 1B). In-line probing^33,34^ analysis reveals that the RNA structure modulates with increasing concentration of branaplam, with a *K*_D_ of 2.44 ± 1.03 μM (Figure S1).

Unexpectedly, the RNA adopts a different secondary structure than anticipated based on the scaffold design. Whereas P2 and P3 are retained from scaffold S8, a P1 substructure is formed from different regions (Figure 2A). Two new stems called P* and P4 also form. Additionally, the capture oligonucleotide binding site, originally designed to overlap with the “left shoulder” of P1, instead overlaps with P*. Based on in-line probing analysis, we inferred that the structure of P* might be metastable because the nucleotides forming this region appear to be less well-structured relative to those within more stable stems such as P2 or P3. The putative capture oligonucleotide binding site also sustains a nucleotide deletion. Perhaps these changes permit the aptamer to release from the capture oligonucleotide more efficiently in the presence of branaplam relative to the original design.

**Figure 2:**
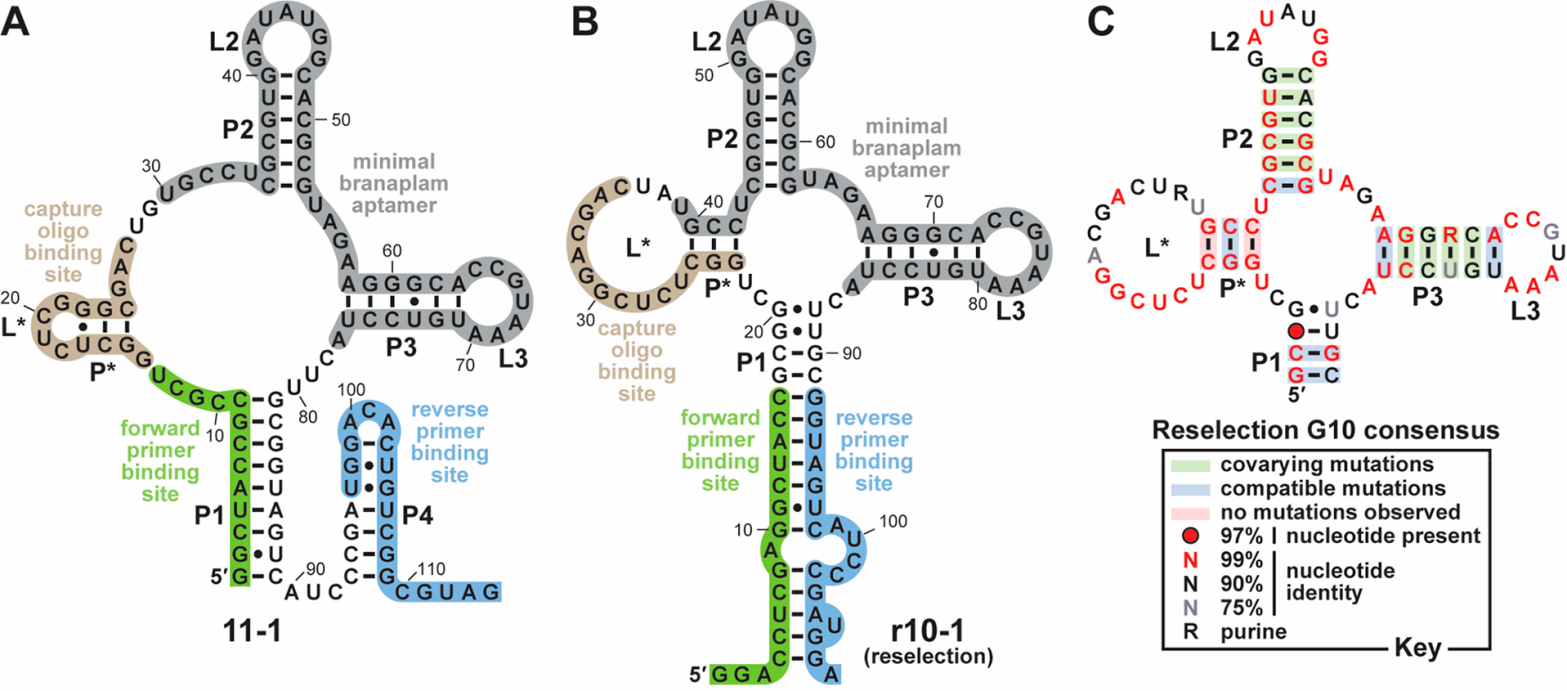
Sequence and secondary structure of branaplam aptamers. A. Sequence and secondary structure model of branaplam aptamer 11–1. This aptamer was the rank 1 candidate by percent abundance in the generation 11 (G11) population, likely descending from scaffold S8. B. Sequence and secondary structure model of branaplam aptamer r10–1. This aptamer was the rank 1 candidate in the generation 10 (G10) population of a branaplam reselection, in which the starting pool was designed based on the core sequence of 11–1 mutagenized at 6% degeneracy per position. C. Consensus model depicting sequencing and secondary structure of the G10 reselection population.

As part of the process to identify a minimal aptamer, a truncated variant called 11–1B was designed that lacks P4. This variant exhibits similar affinity for branaplam with a *K*_D_ of 1.17 ± 0.17 μM (Figure S2). Seeking to identify higher affinity branaplam aptamers, a reselection pool was designed in which the “core” sequence of aptamer 11–1B is mutagenized at 6% degeneracy per position (Figure S3). The sequences of the primer binding sites and the corresponding primers were changed in the reselection pool design so that possible contaminants from the original selection will not be amplified and survive in the reselected population. To improve the likelihood of obtaining higher affinity branaplam aptamers, we decreased the concentration of branaplam used in the reselection by 2 orders of magnitude, from 10 μM to 100 nM. After ten rounds of reselection, the G10 RNA population exhibits a robust elution response to branaplam (Figure S4), indicating that the population has become enriched with molecules that elute with 100 nM branaplam. Sequencing of the G10 reselection population reveals that the top-ranked sequence (r10–1, Figure 2B) comprises 18.4% of the population. In-line probing analysis with radiolabeled r10–1 RNA shows that the aptamer undergoes structural modulation in response to increasing concentrations of branaplam, with a *K*_D_ of 420 ± 41 nM (Figure S5). This constitutes a 5.8-fold improvement in affinity relative to the original aptamer 11–1. We also evaluated candidates ranked 2 through 4 in abundance from the G10 reselection population (r10–2 through r10–4). In-line probing analyses reveal that these RNAs are branaplam aptamers with *K*_D_ values in the range of ∼600 nm to ∼1 μM (Figures S6–S8). Due to the high level of sequence similarity, we consider these molecules to be part of the same class of branaplam aptamers, with r10–1 being the best performing aptamer of those tested.

Our previously reported computational method was used to generate a consensus sequence and secondary structure model for this aptamer class using the sequencing data from the G10 reselection population as an artificial phylogeny (Figure 2C).^24,25^ Notably, the metastable P* appears to have undergone a structural rearrangement relative to the original aptamer 11–1 (Figure 2A). P1 also contains two additional base pairs in the reselected aptamer r10–1 relative to the original 11–1. These changes decrease the lengths of the junction regions except for that between P2 and P3 in aptamers enriched during the reselection.

Next, we sought to improve the affinity of the branaplam aptamer by rational design. We first hypothesized that the loop enclosed by P* (called L*) is not required for binding branaplam despite containing several bases conserved at >99% nucleotide identity (Figure 2C). We reasoned that the nucleotide conservation in L* results from overlap with the capture oligonucleotide binding site, rather than the need to form a binding pocket. To evaluate this, we designed the variant r10–1C, in which L* is replaced with a UUCG tetraloop, which is known to aid the stability of its adjoining base-paired region.^35^ The excess 5′ and 3′ flanking sequences are also trimmed in this construct. In-line probing analysis with r10–1C reveals that the aptamer retains the ability to bind branaplam with a *K*_D_ of 400 ± 43 nM (Figure S9). To confirm that the branaplam-dependent modulation observed is due to a specific binding interaction, and not due to nonspecific interactions, we prepared mutant r10–1C-M1, in which the two pseudoknot-forming G nucleotides present in L2 are mutated to two A nucleotides. In-line probing analysis reveals that this variant rejects branaplam at concentrations up to 10 μM (Figure S9). These results also provide biochemical evidence that the pseudoknot from the Guanine-I aptamer scaffold is maintained in the branaplam aptamer.

Subsequently, a series of truncated constructs called r10–1G through r10–1Q were prepared and assayed by in-line probing for ligand-binding function (Figures S10–S16). The shortest sequence that retains branaplam binding is r10–1N. At just 48 nucleotides in length, r10–1N binds branaplam with a *K*_D_ of 150 ± 26 nM (Figure 3). This RNA retains paired elements P2 and P3 as well as the pseudoknot between L2 and L3 from the Guanine-I scaffold, but, strikingly, lacks P1. The natural aptamer from the Guanine-I riboswitch class requires all three paired elements (P1, P2, and P3) to bind guanine, and crystallographic data has shown that P1 undergoes structural modulation upon binding the target ligand.^36^ In contrast, P1 is not essential for the branaplam aptamer, and constructs without P1 appear to have improved branaplam affinity. After identifying the minimal aptamer r10–1N, we noted that a near-identical sequence occurs in both the original aptamer 11–1 and the reselected aptamer r10–1 (Figure 2). Thus, surprisingly, the reselection did not appear to yield candidates with mutations in the putative ligand-binding site. Instead, the reselection likely improved upon the ability to fold reliably into a productive conformation.

**Figure 3:**
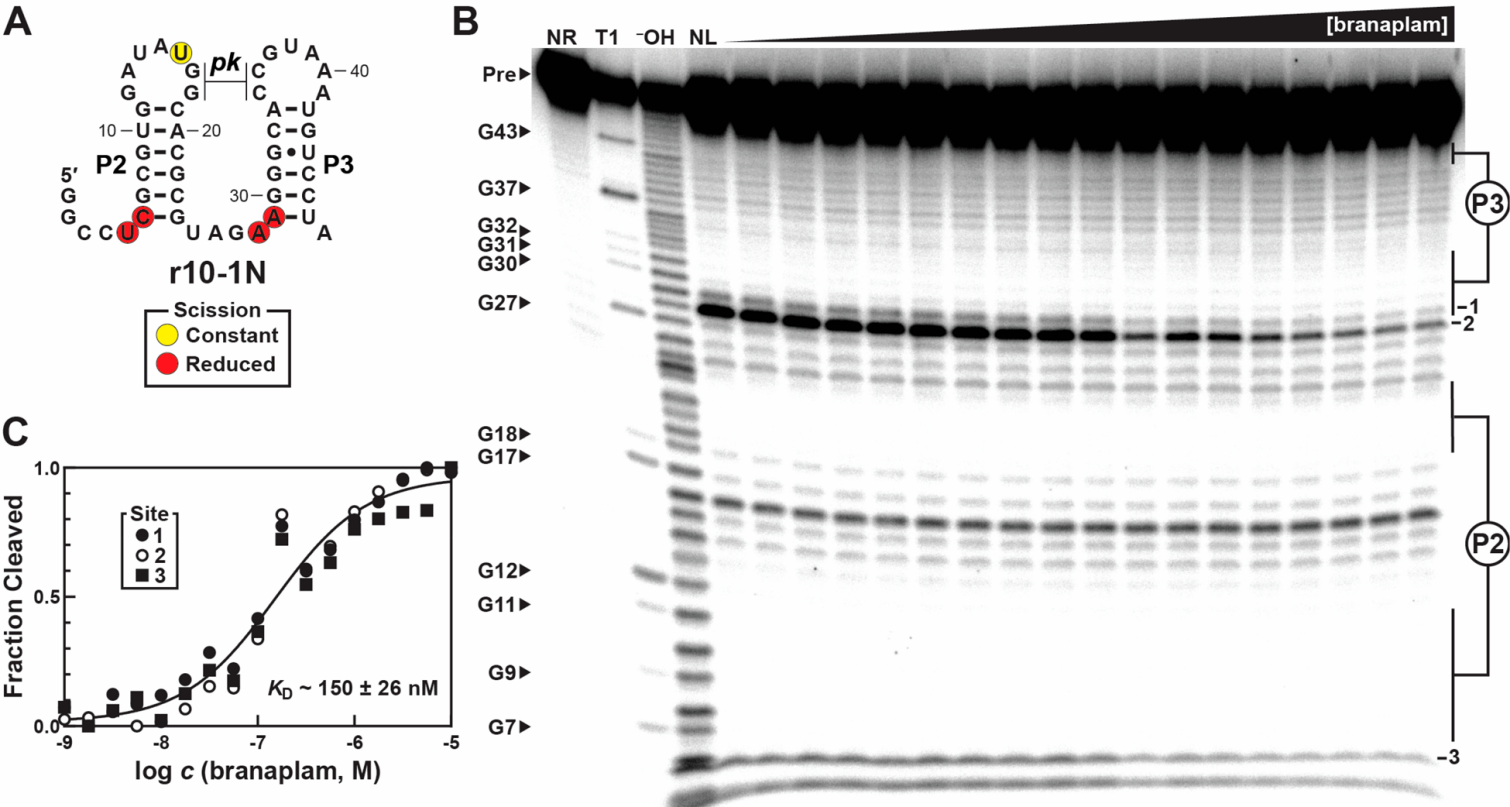
In-line probing reveals secondary structure and binding affinity of branaplam aptamer r10–1N. A. Secondary structure of the r10–1N branaplam aptamer as inferred by in-line probing analysis. A putative pseudoknot is indicated by the regions labeled *pk*. Nucleotides with scissile phosphoester linkages are encircled in yellow or red, corresponding respectively to constant or reduced scission with increasing concentrations of branaplam. B. Autoradiogram depicting in-line probing analysis with 5′ ^32^P-labeled r10–1N RNA incubated with increasing concentrations of branaplam, ranging from 10^–9^ M to 10^–5^ M at quarter-log intervals. Other lanes: no reaction (NR), T1 (treatment with RNase T1, which cleaves at every G nucleotide), ^–^OH (partial alkaline digestion, which cleaves at every nucleotide), and NL (no ligand). Modulating sites 1, 2, and 3 are annotated on the right as well as paired elements P2 and P3. C. Plot of the logarithm of branaplam concentration vs fraction of RNA cleaved at modulating sites 1, 2, and 3. Data were obtained by densitometric analysis of the autoradiogram shown in B. The error associated with the *K*_D_ value is standard error of the mean determined by goodness of fit to a sigmoidal curve (*n* = 1).

Moreover, aptamers 11–1 and r10–1 perform as structure-switching devices with distinct ligand-bound and capture oligonucleotide-bound states. The structural dynamism required to perform as a structure-switching device is expected to erode affinity because the ligand-binding site is in an alternative conformation.^38^ Indeed, allosteric self-cleaving ribozymes typically have larger apparent *K*_D_ values relative to their respective aptamers.^39,40^ The order of magnitude improvement in affinity observed with r10–1N relative to 11–1 is likely due to a reversal of this process.

Specificity is an important characteristic of aptamers expected to function in cells, where thousands of biomolecules are present, some with possible chemical similarity to the target ligand. To assess the specificity of the branaplam aptamer, we investigated the ability of a truncated aptamer to bind various compounds that represent “fragments” of branaplam. In-line probing shows that there is no detectable modulation with any of these fragments at the concentrations tested, up to 10 μM (Figure S17). These results suggest that the aptamer uses multiple molecular recognition contacts with its ligand that are unlikely to be displayed by other compounds naturally present in cells.

We identified an additional candidate, 11–19, in the sequenced G11 population from the original selection whose structure modulates with increasing branaplam concentrations (Figure S18). Based on sequence alignment, 11–19 likely originates from scaffold S10. A truncated variant of this candidate, 11–19A, was assayed by in-line probing and was found to have a *K*_D_ of 846 ± 127 nM (Figure S19). Similarly, a separate selection was performed using a scaffold derived from a TPP (thiamin pyrophosphate) riboswitch aptamer (Figure S20).^37,41^ After 14 rounds of selection, candidate t14–4 was identified, which was assayed by in-line probing and found to have a *K*_D_ of 2.27 ± 0.15 μM (Figure S21). These candidates offer additional opportunities for branaplam aptamer development.

Aptamers with high affinity for their ligand might help to reduce off-target effects of the drug because a nontoxic dose can be administered that still triggers aptamer binding. In clinical trials for Huntington’s Disease, branaplam was administered as 28-mg, 56-mg, 112-mg, 154-mg oral solutions depending on the trial arm.^42^ Assuming the average blood volume in humans is 5 L, a rough estimate of the internal concentration of branaplam used in clinical trials is 14 μM at the smallest dose (28 mg). This value is approximately 100 times greater than the *K*_D_ of the r10–1N branaplam aptamer. It is important to note though that this estimate does not represent the concentration of branaplam inside of a cell, which is difficult to determine.

In a recent report, nuclear magnetic resonance (NMR) was used to show that branaplam binds to the interface between U1 small nuclear ribonucleoparticle (snRNP) and an A_–1_ bulged 5′-splice site.^43^ The r10–1N branaplam aptamer reported here does not exhibit sequence similarity to this natural binding site, suggesting that the molecular recognition determinants are different.

In summary, we identified and validated an RNA aptamer for branaplam that recognizes the target ligand with high affinity and specificity. Using a combination of structure probing and rational design, we have identified a minimal aptamer that retains branaplam binding. Remarkably, the aptamer is only 48 nucleotides long and repurposes structural elements of a natural guanine aptamer. This aptamer could be further developed into a branaplam-responsive riboswitch, which could be employed to regulate expression of a therapeutic transgene in response to branaplam. This process could perhaps be facilitated by exploiting the structure-switching features of the branaplam aptamer originally isolated from in vitro selection. The high affinity of this branaplam aptamer might allow for low concentrations of branaplam to be administered for this repurposed use, which would in turn decrease the potential for off-target effects. We also present two additional aptamer candidates which offer further opportunities for branaplam aptamer development. Future studies are needed to investigate the potential of these RNAs to regulate gene expression in response to branaplam, with potential applications in human gene therapy.

## Supporting Information Available

Materials, Experimental Section, Supplementary Figures (PDF)
