# Supplementary material for: Engineered Branaplam Aptamers Exploit Structural Elements from Natural Riboswitches
Source: ACS Chem Biol. 2024 Jul 2;19(7):1447–52. doi: 10.1021/acschembio.4c00358 (PMC11267568; doi:10.1021/acschembio.4c00358)
Supplement: Supplementary file 1 — cb4c00358_si_001.pdf [file cb4c00358_si_001.pdf]

Supporting Information

Engineered branaplam aptamers that exploit structural elements from natural riboswitches

Michael G. Mohsen, Matthew K. Midy, Aparaajita Balaji, and Ronald R. Breaker*
*Corresponding author

Table of Contents

MATERIALS.......... 3
Chemicals.......... 3
Enzymes.......... 3
List of Custom Oligodeoxynucleotides.......... 3
Instrumentation.......... 7
Other Materials.......... 7
EXPERIMENTAL SECTION.......... 8
Synthesis of S7-S12 G0 Pool.......... 8
In vitro Selection (Scaffolds S7 through S12).......... 8
In vitro Selection (TPP Scaffold).......... 9
Branaplam Reselection.......... 9
Elution Profiling.......... 9
In-line Probing.......... 9
Next-generation Sequencing.......... 10
Bioinformatics.......... 10
SUPPLEMENTARY FIGURES.......... 11
Figure S1. In-line probing of aptamer 11-1 with branaplam.......... 11
Figure S2. In-line probing of truncated aptamer 11-1B with branaplam.......... 12
Figure S3. Branaplam reselection pool.......... 13
Figure S4. Elution profile of the G10 reselection population.......... 14
Figure S5. In-line probing of r10-1 with branaplam.......... 15
Figure S6. In-line probing of r10-2 with branaplam.......... 16
Figure S7. In-line probing of r10-3 with branaplam.......... 17
Figure S8. In-line probing of r10-4 with branaplam.......... 18
Figure S9. In-line probing of r10-1C and r10-1C-M1 with branaplam.......... 19

Figure S10. In-line probing of r10-1G with branaplam....................20
Figure S11. In-line probing of r10-1H with branaplam....................21
Figure S12. In-line probing of r10-1I with branaplam....................22
Figure S13. In-line probing of r10-1J with branaplam....................23
Figure S14. In-line probing of r10-1K with branaplam....................24
Figure S15. In-line probing of r10-1L through r10-1P with branaplam....................25
Figure S16. In-line probing of r10-1Q with branaplam....................26
Figure S17. In-line probing of r10-1H with branaplam derivatives....................27
Figure S18. In-line probing of 11-19 with branaplam....................28
Figure S19. In-line probing of 11-19A with branaplam....................29
Figure S20. A scaffold inspired by a natural TPP riboswitch aptamer....................30
Figure S21. In-line probing of t14-4 with branaplam....................31
REFERENCES....................32

MATERIALS

Chemicals

Branaplam was obtained from MedChemExpress. Branaplam derivatives 2,2,6,6-tetramethyl-4-piperidinol (**1**), 3-chloro-6-((2,2,6,6-tetramethylpiperidin-4-yl)oxy)pyridazine (**2**), 2-(6-chloro-3-pyridazinyl)phenol (**3**), 6-chloro-3-hydroxypyridazine (**4**), and 3-(1H-pyrazol-4-yl)phenol (**5**) were obtained from Sigma Aldrich. Custom oligodeoxynucleotides were purchased from IDT, Sigma Aldrich, or the Keck Oligonucleotide Synthesis facility. Custom double stranded DNA (gBlocks) were purchased from IDT.

Enzymes

Taq DNA polymerase and T4 polynucleotide kinase were obtained from New England Biolabs. SuperScript III and TURBO DNase were obtained from Invitrogen. rAPid alkaline phosphatase was obtained from Roche. RNase T1 was obtained from Thermo Scientific. All enzymes were used with the provided buffers and recommended reaction conditions following the manufacturer's instructions, unless stated otherwise. T7 RNA polymerase was purified in-house.

List of Custom Oligodeoxynucleotides

Name	Sequence (5′→3′)	Length (nt)	Notes
FWD-N8	TAATACGACTCACTATAGGCTACCGCCGCTGG<br>GCTCTCGGGACGACNNNNNNNNGCGTGGATAT<br>GGCACGC	71	
FWD-N11	TAATACGACTCACTATAGGCTACCGCCGCTGG<br>GCTCTCGGGACGACNNNNNNNNNNNGCGTGGA<br>TATGGCACGC	74	
FWD-N6	TAATACGACTCACTATAGGCTACCGCCGCTGG<br>GCTCTCGGGACGACNNNNNNGCGTGGATATGG<br>CACGC	69	
REV-N11-N6	CTACGCCGACAGTGTCCATCGGGACGACNNNN<br>NNGGACATTTACGGTGCCCNNNNNNNNNNNGC<br>GTGCCATATCCAC	77	
REV-N6-N11	CTACGCCGACAGTGTCCATCGGGACGACNNNN<br>NNNNNNNGGACATTTACGGTGCCCNNNNNNGC<br>GTGCCATATCCAC	77	

REV-N8-N6	CTACGCCGACAGTGTCCATCGGGACGACNNNNNNNNGGACATTTACGGTGCCCNNNNNNGCGTGCCATATCCAC	74	
REV-N6-N8	CTACGCCGACAGTGTCCATCGGGACGACNNNNNNGGACATTTACGGTGCCCNNNNNNNNGCGTGCCATATCCAC	74	
REV-N11-N8	CTACGCCGACAGTGTCCATCGGGACGACNNNNNNNNGGACATTTACGGTGCCCNNNNNNNNNNNGCGTGCCATATCCAC	79	
REV-N8-N11	CTACGCCGACAGTGTCCATCGGGACGACNNNNNNNNNNNGGACATTTACGGTGCCCNNNNNNNNGCGTGCCATATCCAC	79	
S7-S12 FWD PCR Primer	TAATACGACTCACTATAGGCTACCGCCGCTG	31	
S7-S12 REV PCR Primer	CTACGCCGACAGTGTCCA	18	
S7-S12 Capture Oligonucleotide	GTCGTCCCGAGAGCCATA	18	3′ TEG-biotin modification
11-1	TAATACGACTCACTATAGGCTACCGCCGCTGGCTCTCGGGCGACTGTGCCTCGCGTGGATATGGCACGCGTAGAAGGGCACCGTAAATGTCCTACTTGCGGTAGTCATCCCGATGGACACTGTCGGCGTAG	131	gBlock
11-1B	TAATACGACTCACTATAGGCTACCGCCGCTGGCTCTCGGGCGACTGTGCCTCGCGTGGATATGGCACGCGTAGAAGGGCACCGTAAATGTCCTACTTGCGGTAGTC	106	FWD+REV
RS FWD PCR Primer	TAATACGACTCACTATAGGACCTCGAGGCTACC	33	
RS REV PCR Primer	TCCATCGGGATGACTACC	18	
RS Template	TCCATCGGGATGACTACC6755685665758885766867778878576768677585877576	107	5 = 94:2:2:2<br>6 = 2:94:2:2<br>7 = 2:2:94:2

	76566757568767776565677567667GGT<br>AGCCTCGAGGT		8 = 2:2:2:94<br>(A:G:C:T)
r10-1	TAATACGACTCACTATAGGACCTCGAGGCTAC<br>CGCGGCTGGCTCTCGGACGACTATGCCTCGCG<br>TGGATATGGCACGCGTAGAAGGGCACCGTAAA<br>TGTCCTACTTGCGGTAGTCATCCCGATGGA	126	gBlock
r10-2	TAATACGACTCACTATAGGACCTCGAGGCTAC<br>CGCAGCTGGCTCTCGGACGACTATGCCTCGCG<br>TGGATATGGCACGCGTAGAAGGGCACCGTAAA<br>TGTCCTACTTGCGGTAGTCATCCCGATGGA	126	gBlock
r10-3	TAATACGACTCACTATAGGACCTCGAGGCTAC<br>CGCGGCTGGCTCTCGGACGACTGTGCCTCGCG<br>TGGATATGGCACGCGTAGAAGGACACCGTAAA<br>TGTCCTACTTGCGGTAGTCATCCCGATGGA	126	gBlock
r10-4	TAATACGACTCACTATAGGACCTCGAGGCTAC<br>CGCGGCTGGCTCTCGGACGACTATGCCTCGCG<br>TGGATATGGCACGCGTAGAAGGGCACCGTAAA<br>TGCCCTACTTGCGGTAGTCATCCCGATGGA	126	gBlock
r10-1C	TAATACGACTCACTATAGGCTACCGCGGCTGG<br>CTTCGGCCTCGCGTGGATATGGCACGCGTAGA<br>AGGGCACCGTAAATGTCCTACTTGCGGTAGTC	96	FWD+REV
r10-1C-M1	TAATACGACTCACTATAGGCTACCGCGGCTGG<br>CTTCGGCCTCGCGTGGATATAACACGCGTAGA<br>AGGGCACCGTAAATGTCCTACTTGCGGTAGTC	96	FWD+REV
r10-1G	TAATACGACTCACTATAGGCTGGCTTCGGCCT<br>CGCGTGGATATGGCACGCGTAGAAGGGCACCG<br>TAAATGTCCTACTT	78	FWD+REV
r10-1H	TAATACGACTCACTATAGGCCTCGCGTGGATA<br>TGGCACGCGTAGAAGGGCACCGTAAATGTCCT<br>ACTT	68	FWD+REV
r10-1I	TAATACGACTCACTATAGGCGCGTGGATATGG<br>CACGCGTAGAAGGGCACCGTAAATGTCCT	61	FWD+REV
r10-1J	TAATACGACTCACTATAGGCCTCGCGTGGATA<br>TGGCACGCGTAGAAGGGCACCGTAAATGTCCT	64	FWD+REV

r10-1K	TAATACGACTCACTATAGGCGCGTGGATATGGCACGCGTAGAAGGGCACCGTAAATGTCCTACTT	65	FWD+REV
r10-1L	TAATACGACTCACTATAGGCCTCGCGTGGATATGGCACGCGTAGAAGGGCACCGTAAATGTCCTACT	67	FWD+REV
r10-1M	TAATACGACTCACTATAGGCCTCGCGTGGATATGGCACGCGTAGAAGGGCACCGTAAATGTCCTAC	66	FWD+REV
r10-1N	TAATACGACTCACTATAGGCCTCGCGTGGATATGGCACGCGTAGAAGGGCACCGTAAATGTCCTA	65	FWD+REV
r10-1O	TAATACGACTCACTATAGGCTCGCGTGGATATGGCACGCGTAGAAGGGCACCGTAAATGTCCTACTT	67	FWD+REV
r10-1P	TAATACGACTCACTATAGGTCGCGTGGATATGGCACGCGTAGAAGGGCACCGTAAATGTCCTACTT	66	FWD+REV
r10-1Q	TAATACGACTCACTATAGCCTCGCGTGGATATGGCACGCGTAGAAGGGCACCGTAAATGTCCTA	64	FWD+REV
11-19	TAATACGACTCACTATAGGCTACCGCCGCTGGGCTCTCGGGACGACACTGAGGGAACGCGTGGATATGGCACGCAACAGCGGGCACCGTAAATGTCCTGGCGGTAGTCGACCCGATGGACACTGTCGGCGTAG	133	gBlock
11-19A	TAATACGACTCACTATAGGCTACCGCCGCTGGGCTCTCGGGACGACACTGAGGGAACGCGTGGATATGGCACGCAACAGCGGGCACCGTAAATGTCCTGGCGGTAGTC	108	FWD+REV
TPP Template FWD	TAATACGACTCACTATAGGCTGATCTCCCATCGGNNNGCCCTTCTGCGTGAAGGC	56	
TPP Template REV	GCGTGCGTCGTAGTATCTCCCATNNNNNNNNCTGGCATTATCCAGNNNNNNNNNNNCGGNNNNNNNNNNGCCTTCACGCAGAAG	85	

TPP FWD PCR primer	TAATACGACTCACTATAGGCTGATCTCCCATCGG	34	
TPP REV PCR primer	GCGTGCGTCGTAGTATCTCC	20	
TPP Capture Oligonucleotide	TGGGAGATCAGC	12	3′ TEG-biotin modification
t14-4	GCGTGCGTCGTAGTATCTCCATACCACTAACTGGCATTATCCAGTTTCTACACTCCGGCATTAGTACCGCCTTCACACAGAAGGGCATACCGATGGGAGATCAGCC	106	dsDNA prepared by primer extension with TPP FWD PCR primer

Note: for oligonucleotides designated “FWD+REV” the forward sequence (as shown) and the reverse complement sequence were both ordered and used for experiments.

Instrumentation

Eppendorf Nexus Thermal Cycler (Mastercycler Nexus Gx2, Mastercycler Nexus Gx2e, Mastercycler Nexus Gx2e) was used for thermal cycling, *e.g.*, PCR. Bio-Rad CFX Opus RT-PCR System (CFX Opus 96) was used for quantitative PCR (qPCR). Typhoon FLA9500 was used for phosphor imaging. Gel Doc Go Imaging System (Bio-Rad) was used for fluorescence imaging of agarose gels stained with ethidium bromide. NanoDrop 8000 (Thermo Fisher) was used to quantitate the concentration of nucleic acid solutions.

Other Materials

Pierce Streptavidin Agarose (#20353) was obtained from Thermo Scientific. Micro Bio-Spin Chromatography Columns (#7326204) were obtained from Bio-Rad.

EXPERIMENTAL SECTION

Synthesis of S7-S12 G0 Pool

For each of the six scaffolds S7-S12, the corresponding forward and reverse oligonucleotides (100 pmol each) for each scaffold were mixed as shown below:

**Scaffold**	**Forward**	**Reverse**
S7 (N8-N11-N6)	FWD-N8	REV-N11-N6
S8 (N8-N6-N10)	FWD-N8	REV-N6-N11
S9 (N11-N8-N6)	FWD-N11	REV-N8-N6
S10 (N11-N6-N8)	FWD-N11	REV-N6-N8
S11 (N6-N11-N8)	FWD-N6	REV-N11-N8
S12 (N6-N8-N11)	FWD-N6	REV-N8-N11

To each of the six solutions, 5 µL 10 mM dNTPs and deionized, sterile H_2O (dH_2O) were added to a final volume of 37.5 µL. Each solution was heated to 90 °C for 1 min, and subsequently allowed to cool to room temperature on the benchtop (~3 min). Then, 10 µL 5x First Strand buffer (provided by the manufacturer), 5 µL 0.1 M DTT, and 2 µL SuperScript III RT were added to each solution. Primer extension was performed by incubating each solution at 55 °C for 1 h. The RT was heat-denatured by incubating at 75 °C for 15 min, but no further purification was performed. 12.5 µL (~25 pmol DNA) from each of these reactions was used in subsequent in vitro transcription reactions, which also contained 55 µL dH_2O, 10 µL 10x transcription buffer [150 mM $MgCl_2$, 20 mM spermidine, 500 mM Tris (pH 7.5 at ~20 °C), and 50 mM DTT], 20 µL 10 mM NTPs, and 2.5 µL T7 RNA polymerase. Each reaction was incubated at 37 °C for 2 h. To deplete template DNA, 1 µL Turbo DNase was added and each reaction was incubated at 37 °C for 10 min. The resulting RNA pool was purified by denaturing (8 M urea) 10% polyacrylamide gel electrophoresis. The band containing the target RNA was excised from the gel, excluding the top and bottom 20% of the band. RNA was eluted from the gel by crushing and soaking in buffer [0.4 M NaCl, 10 mM Tris, 1 mM EDTA (pH 8 at ~20 °C)] and subsequently concentrated by precipitation in ethanol.

In vitro Selection (Scaffolds S7 through S12)

In vitro selection was performed as previously described.[1] Briefly, the G0 RNA pool was mixed with a 10x molar excess of capture oligonucleotide in selection buffer (20 mM HEPES, 100 mM KCl, 3 mM NaCl (total [Na^+]: 10 mM), and 1 mM $MgCl_2$, pH 7.5 at ~20 °C) and the resulting solution was incubated at 90 °C for 1 min. The RNA-capture oligonucleotide solution was loaded onto a Micro-Bio spin column loaded with streptavidin-agarose. After stringent washing, a solution

containing 10 μM branaplam (as well as three other target compounds) was loaded onto the column. RNA molecules that eluted in the presence of the target compounds were amplified by reverse-transcription polymerase chain reaction (RT-PCR). This process was repeated 11 times, resulting in the G11 population. During selection rounds 1 through 6, the column was incubated with the target ligand solution for 2.5 min. During selection rounds 7 through 11, the incubation time was shortened to 0.5 min.

In vitro Selection (TPP Scaffold)

The process was performed as described above for scaffolds S7 through S12. 14 rounds of in vitro selection were performed, resulting in the G14 population.

Branaplam Reselection

The branaplam reselection (BRS) template was designed based on the sequence of BRS-G11-R1B with different primer-binding regions. 75 nucleotides between the primer-binding regions were mutagenized at 6% degeneracy. The selection was performed as described above, except that the target solution contained 100 nM branaplam. Ten selection cycles were performed, resulting in the BRS-G10 population.

Elution Profiling

Elution profiles were performed as previously described.[1] Briefly, RNA was synthesized by in vitro transcription with T7 RNA polymerase, desphosphorylated with alkaline phosphatase, and radiolabeled with T4 polynucleotide kinase in the presence of γ-^{32}P-ATP. Elution profiles were performed similarly to the selection. 1 μL of each solution was pipetted onto filter paper and phosphor imaged using a Typhoon scanner.

In-line Probing

In-line probing was performed as previously described.[2] Briefly, 5′-^{32}P-labeled RNA was mixed with the ligand at the specified concentration and 2x in-line probing buffer (20 mM $MgCl_2$, 100 mM KCl, 50 mM Tris-HCl [pH 8.3 at ~20 °C]). After incubating at room temperature for ~48 h, the reaction was quenched by the addition of 2x loading buffer [18 M urea, 20% w/v sucrose, 0.1% w/v sodium dodecyl sulfate, 0.05% w/v bromophenol blue, 0.05% xylene cyanol, 90 mM Tris, 90 mM borate, 1 mM EDTA pH 8.0 at ~20 °C)]. RNase T1, which cleaves at every G nucleotide, was used according to the manufacturer's instructions to generate a "T1 ladder" for each RNA. $^{-}$OH ladders were prepared by treatment with 50 mM sodium bicarbonate at 90 °C for 1 min. The

samples were analyzed by 10% denaturing (8 M urea) PAGE. The resulting gels were dried and subsequently phosphor imaged using a Typhoon scanner. Densitometry was performed with ImageJ software.

Next-generation Sequencing

50 ng of the corresponding DNA population were submitted to the Yale Center for Genomic Analysis. The populations were sequenced at a depth of about 40 million reads. Paired-end reads were sequenced with a read length of 150 base pairs.

Bioinformatics

Computational sequence analysis was performed as described previously.[1] The python script *toTally* was used to calculate percent abundances of unique sequences in each sequenced population.[3] The python script *selfishCluster* was used to generate clusters of similar sequences.[3] CMfinder was used to generate Stockholm files (.sto) containing putative structural information.[4] R2R was used to draw the output of CMfinder.[5]

SUPPLEMENTARY FIGURES

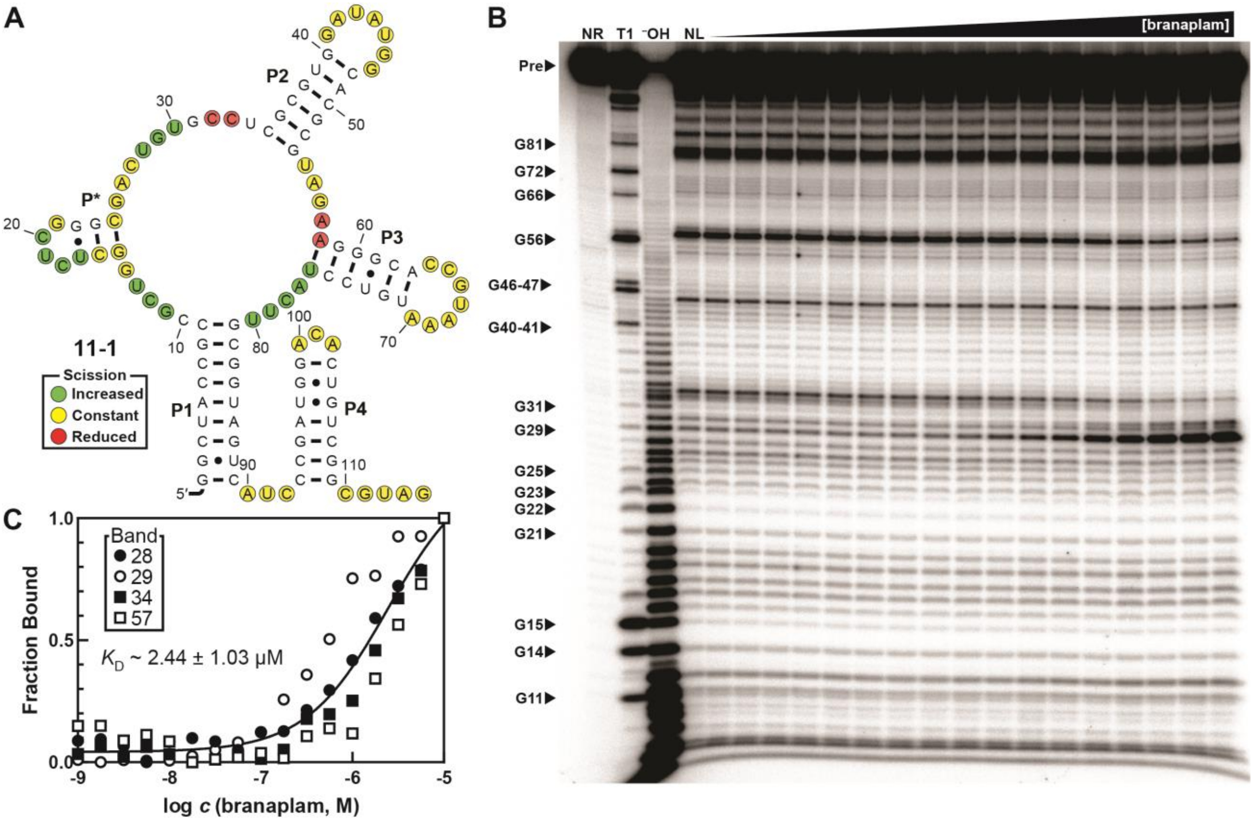

Figure S1. In-line probing of aptamer 11-1 with branaplam

A. Secondary structure diagram of 11-1, which comprised 13.1% of the G11 population in the original selection. **B.** Autoradiogram of a polyacrylamide gel showing the result of in-line probing reactions containing radiolabeled 11-1 RNA and increasing branaplam concentrations ranging from 10^{-9} to 10^{-5} M at quarter-log intervals. **C.** Plot of the logarithm of branaplam concentration vs. fraction of RNA bound. The K_D for this interaction is 2.44 ± 1.03 μM.

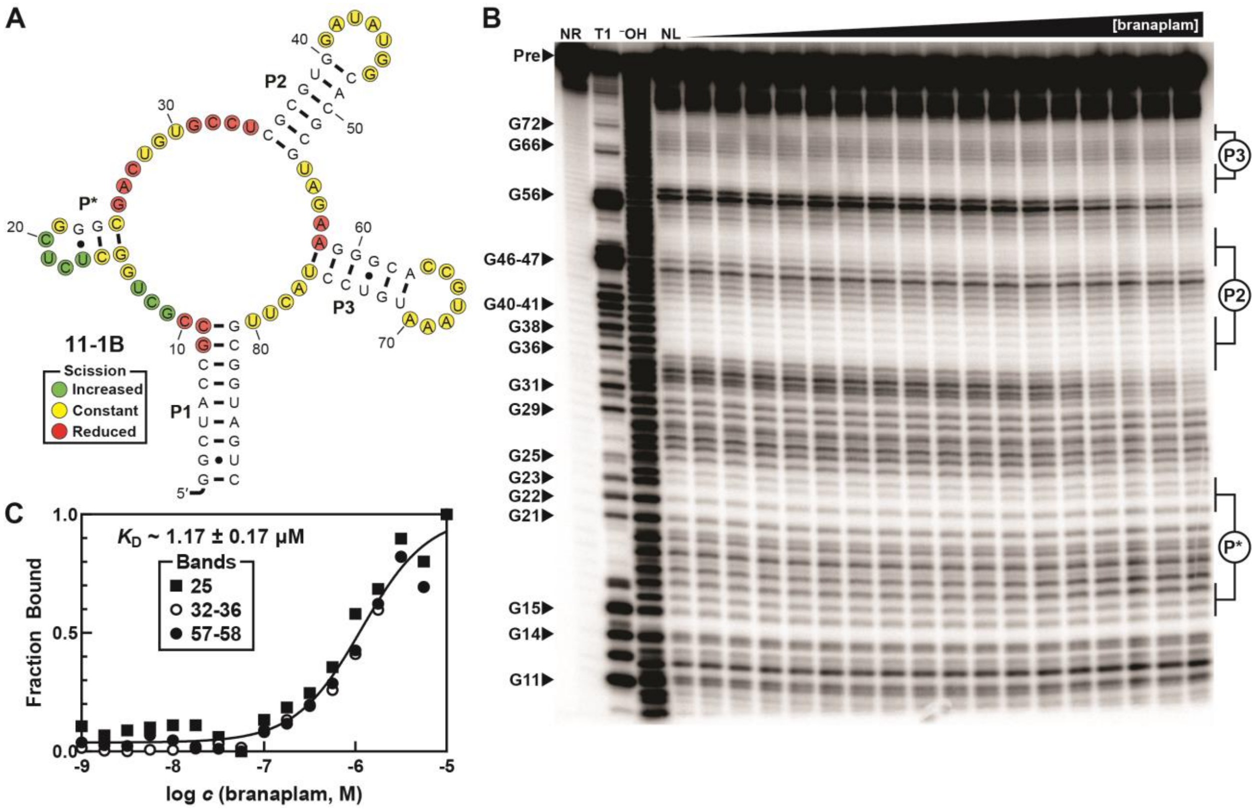

Figure S2. In-line probing of truncated aptamer 11-1B **with branaplam**

A. Sequence and secondary structure model of 11-1B. **B.** Autoradiogram of a polyacrylamide gel showing the result of in-line probing reactions containing radiolabeled 11-1B RNA and increasing branaplam concentrations ranging from 10^{-9} to 10^{-5} M at quarter-log intervals. **C.** Plot of the logarithm of branaplam concentration vs. the fraction of RNA bound. The K_D for this interaction is 1.17 ± 0.17 µM.

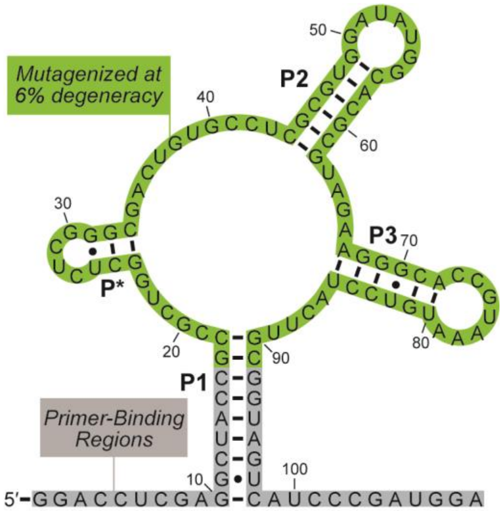

Figure S3. Branaplam reselection pool

RNA pool design for branaplam reselection. The region highlighted in green is mutagenized at 6% degeneracy. The flanking regions highlighted in grey are the primer-binding regions, which were not mutagenized.

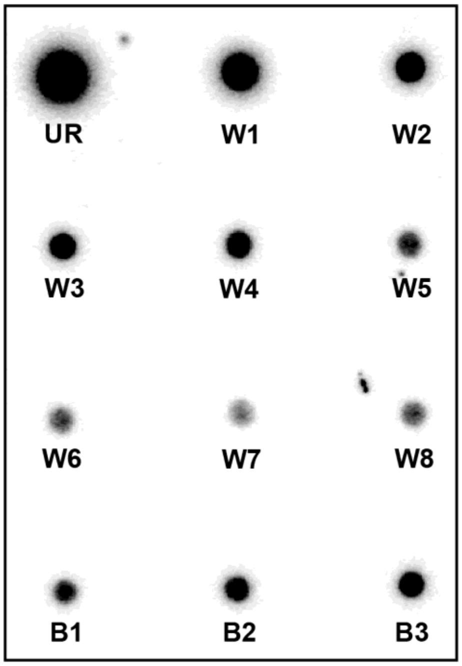

Figure S4. Elution profile of the G10 reselection population

Autoradiogram of an elution profile with radiolabeled G10 reselection population depicting the relative amounts of radioactivity from sequential elutions. Key – UR: unbound RNA, W*n*: wash with selection buffer in which *n* corresponds to the number of washes that have been performed, B*n*: elution with 100 nM branaplam dissolved in selection buffer in which *n* corresponds to the number of elutions that have been performed.

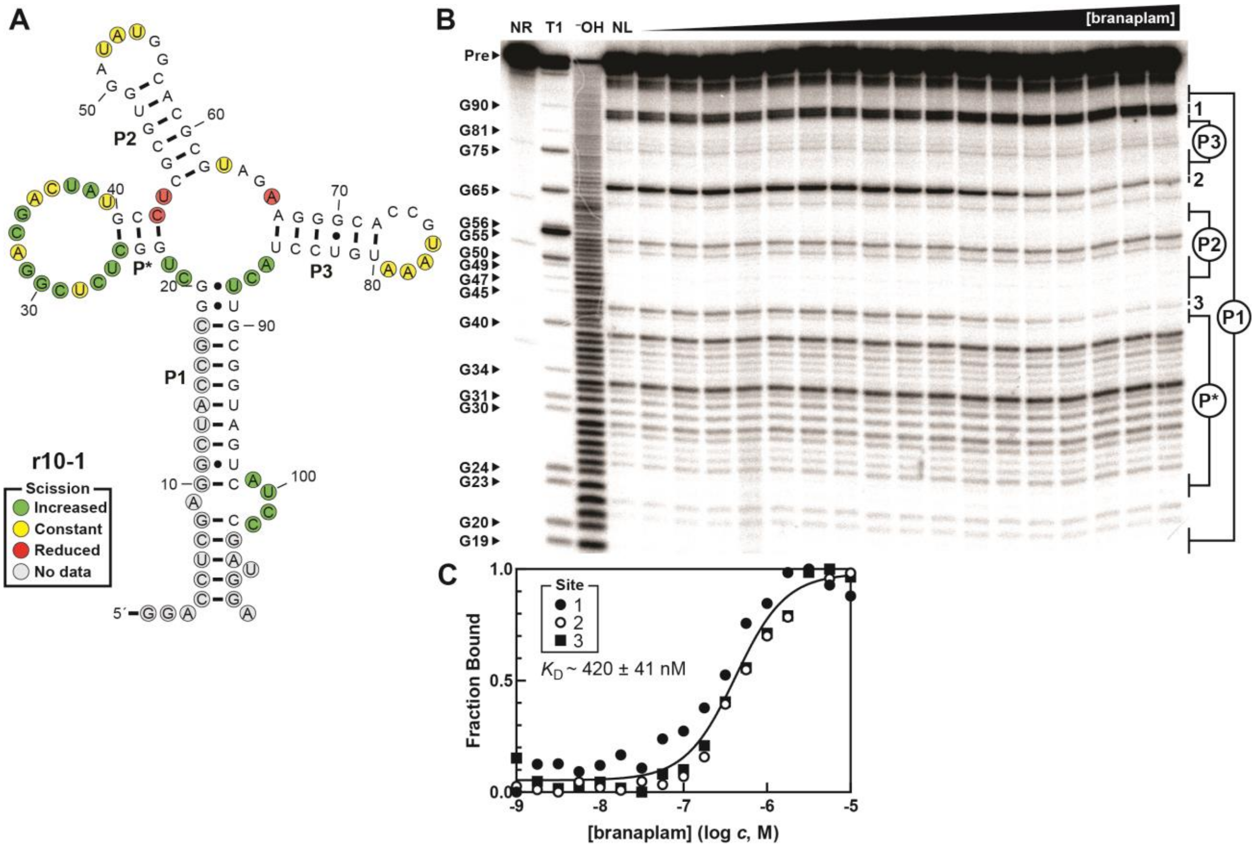

Figure S5. In-line probing of r10-1 with branaplam

A. Sequence and secondary structure model of r10-1 RNA, which comprised 18.4% of the G10 reselection population. **B.** Autoradiogram of in-line probing gel with 5′-^{32}P-labeled r10-1 RNA incubated with increasing branaplam concentrations ranging from 10^{-9} to 10^{-5} M at quarter-log intervals. **C.** Plot of the logarithm of branaplam concentration vs. the fraction of RNA bound. The K_D for this interaction is 420 ± 41 nM.

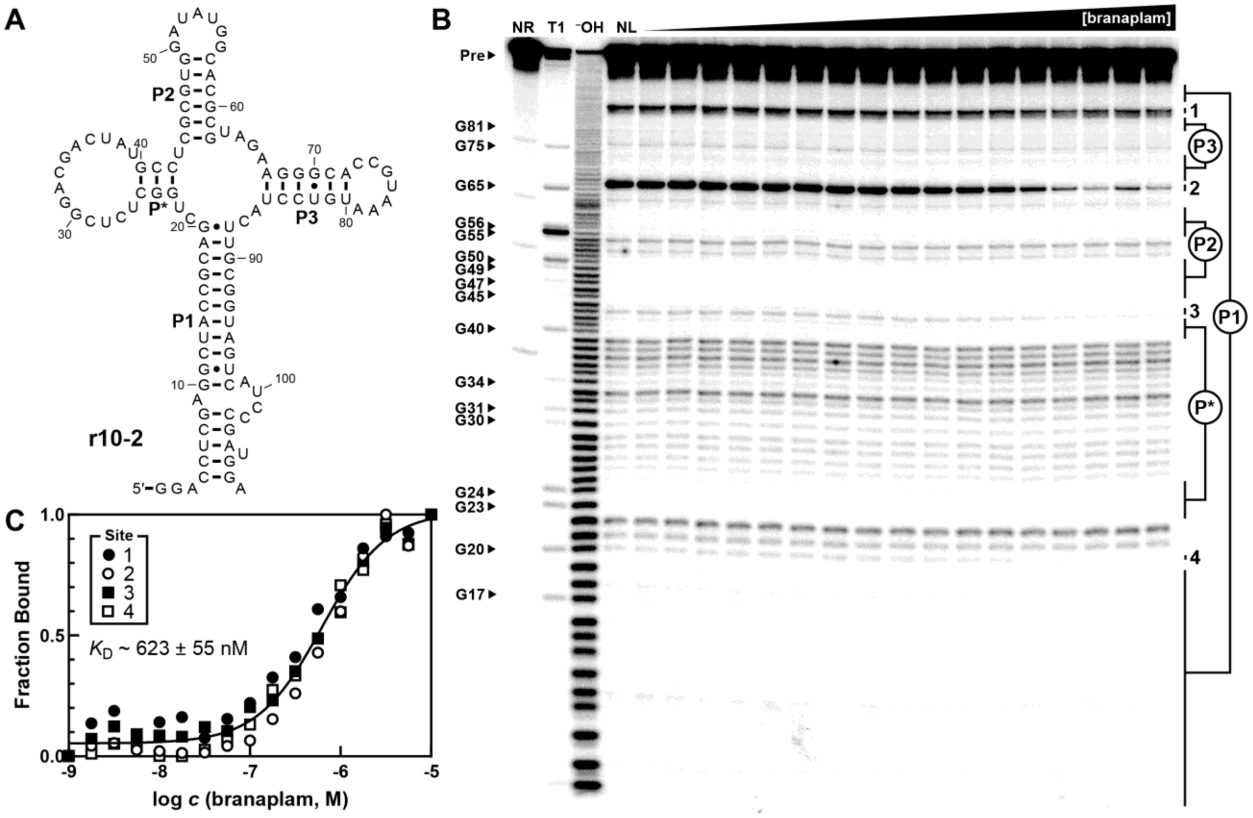

Figure S6. In-line probing of r10-2 with branaplam

A. Sequence and secondary structure model of r10-2 RNA, which comprised 5.2% of the G10 reselection population. **B.** Autoradiogram of in-line probing gel with 5′-^{32}P-labeled r10-2 RNA incubated with increasing branaplam concentrations ranging from 10^{-9} to 10^{-5} M at quarter-log intervals. **C.** Plot of the logarithm of branaplam concentration vs. the fraction of RNA bound. The K_D for this interaction is 623 ± 55 nM.

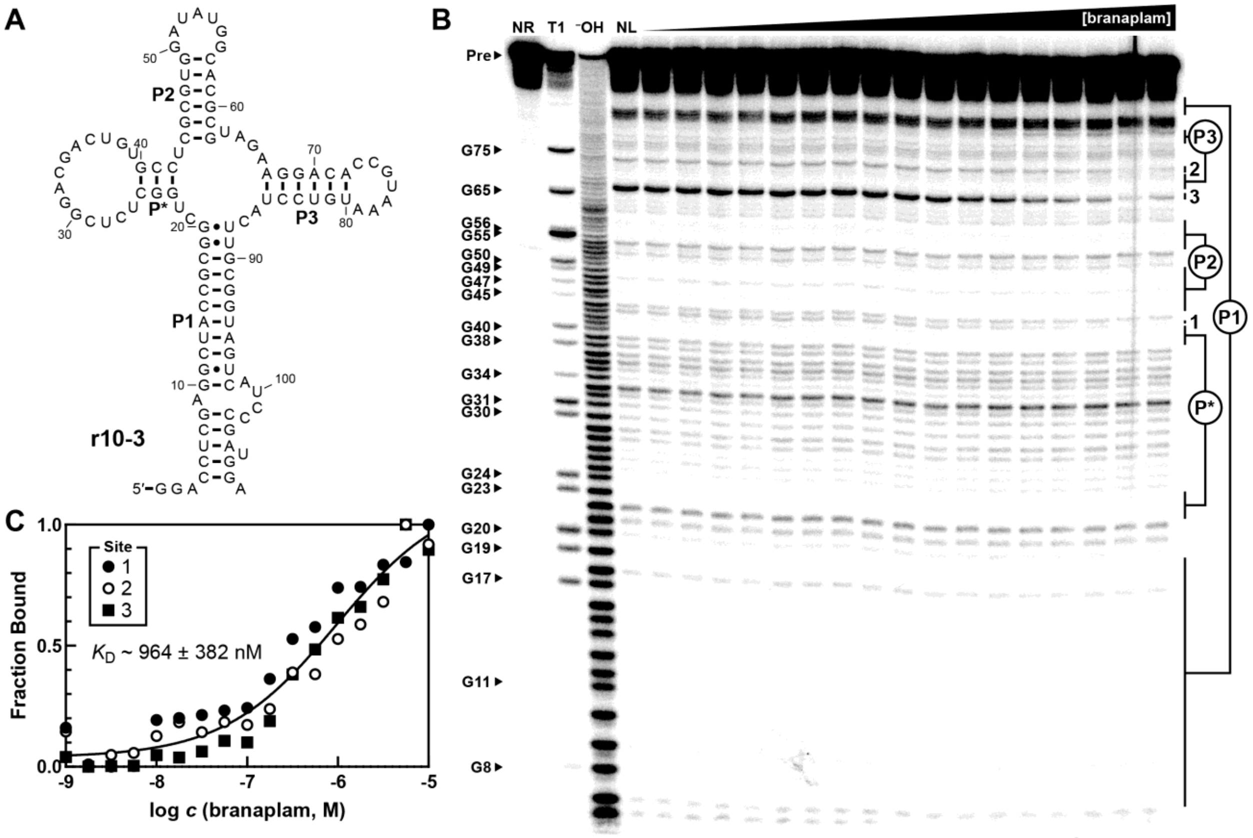

Figure S7. In-line probing of r10-3 with branaplam

A. Sequence and secondary structure model of r10-3 RNA, which comprised 5.1% of the G10 reselection population. **B.** Autoradiogram of in-line probing gel with 5′-^{32}P-labeled r10-1 RNA incubated with increasing branaplam concentrations ranging from 10^{-9} to 10^{-5} M at quarter-log intervals. **C.** Plot of the logarithm of branaplam concentration vs. the fraction of RNA bound. The K_D for this interaction is 964 ± 382 nM.

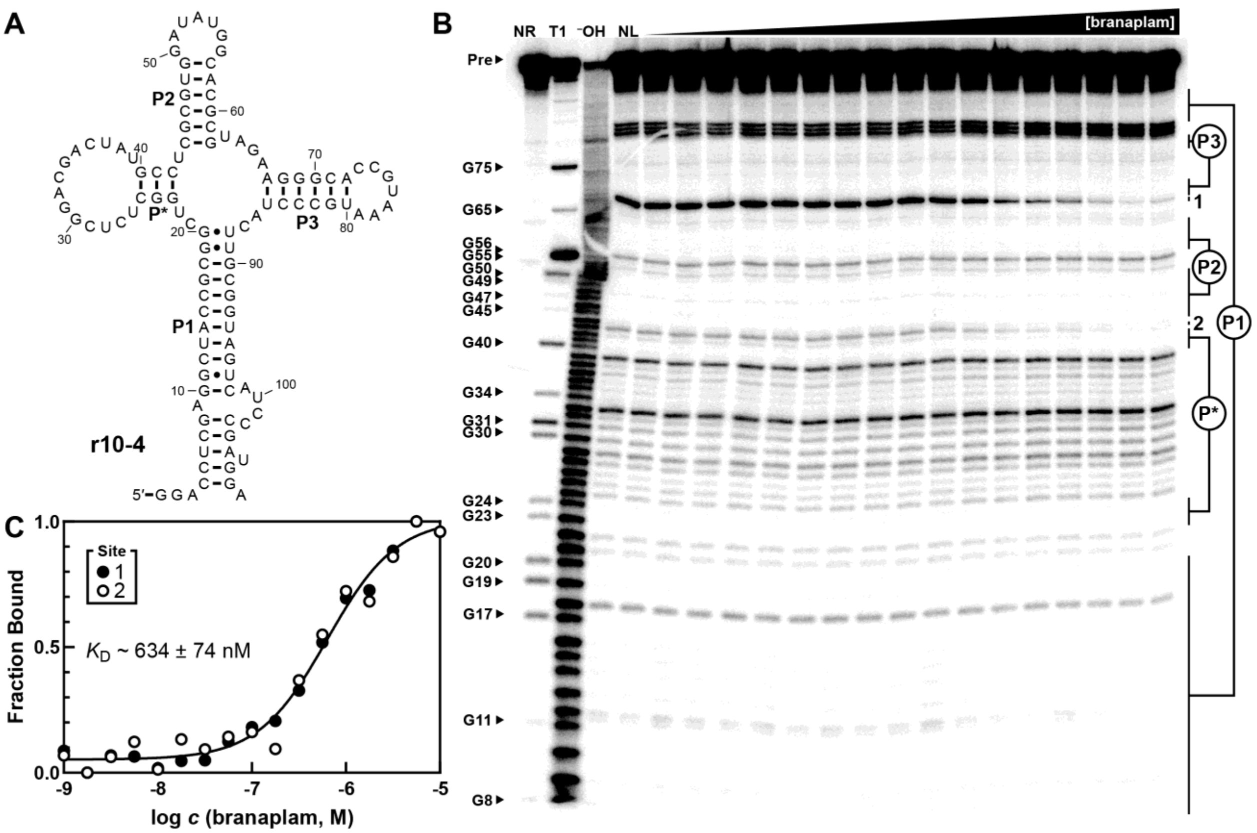

Figure S8. In-line probing of r10-4 with branaplam

A. Sequence and secondary structure model of r10-4 RNA, which comprised 4.1% of the G10 reselection population. **B.** Autoradiogram of in-line probing gel with 5′-^{32}P-labeled r10-4 RNA incubated with increasing branaplam concentrations ranging from 10^{-9} to 10^{-5} M at quarter-log intervals. **C.** Plot of the logarithm of branaplam concentration vs. the fraction of RNA bound. The K_D for this interaction is 634 ± 74 nM.

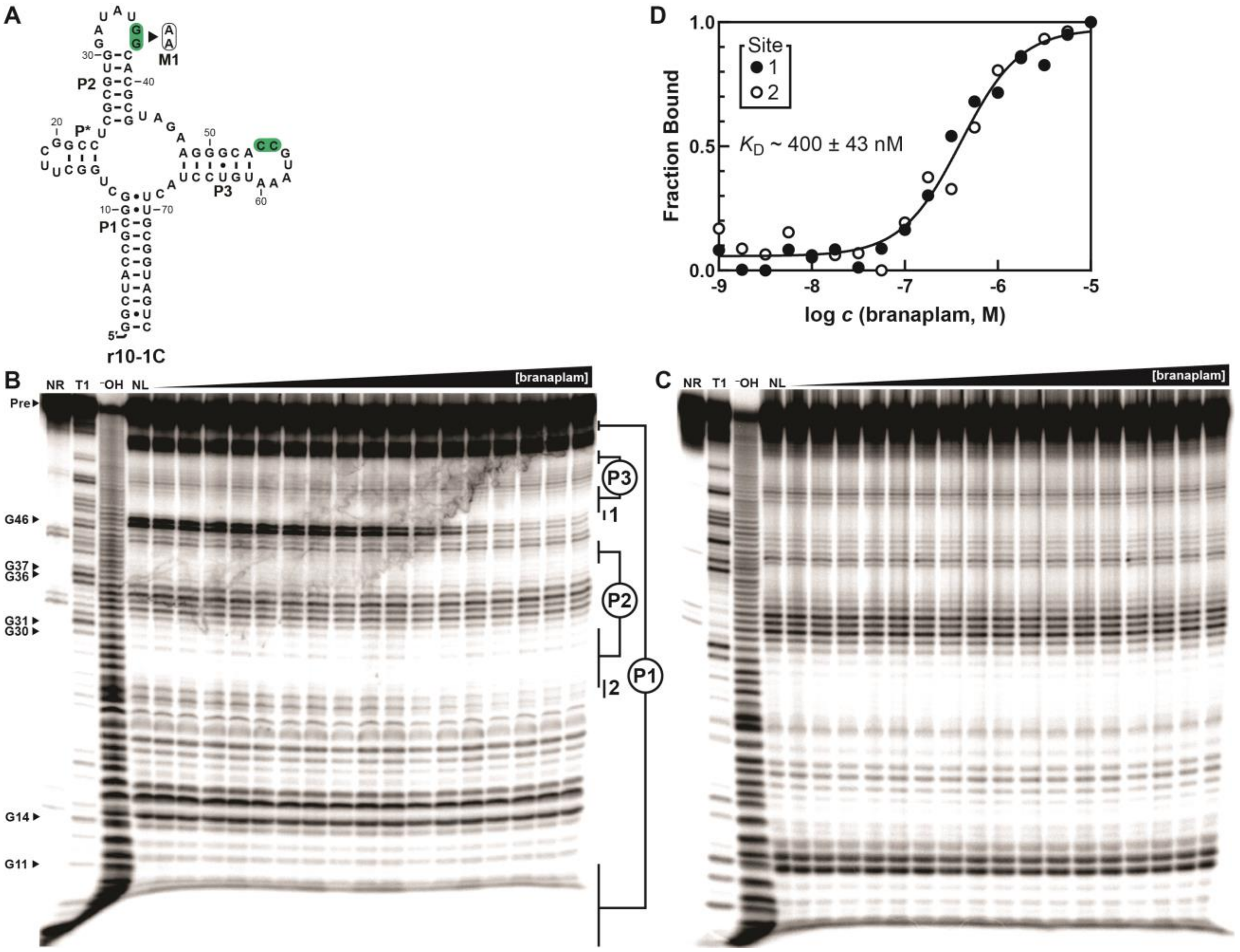

Figure S9. In-line probing of r10-1C and r10-1C-M1 with branaplam

A. Sequence and secondary structure model of r10-1C RNA. The nucleotides highlighted in green indicate the location of a putative pseudoknot. The r10-1C-M1 variant contains two G to A mutations at nucleotides 36 and 37 as shown. **B.** Autoradiogram of in-line probing gel with 5′-^{32}P-labeled r10-1C RNA incubated with increasing branaplam concentrations ranging from 10^{-9} to 10^{-5} M at quarter-log intervals. **C.** Autoradiogram of in-line probing gel with 5′-^{32}P-labeled r10-1C-M1 RNA incubated with increasing branaplam concentrations ranging from 10^{-9} to 10^{-5} M at quarter-log intervals. **D.** Plot of the logarithm of branaplam concentration vs. the fraction of r10-1C RNA bound. The K_D for this interaction is 400 ± 43 nM.

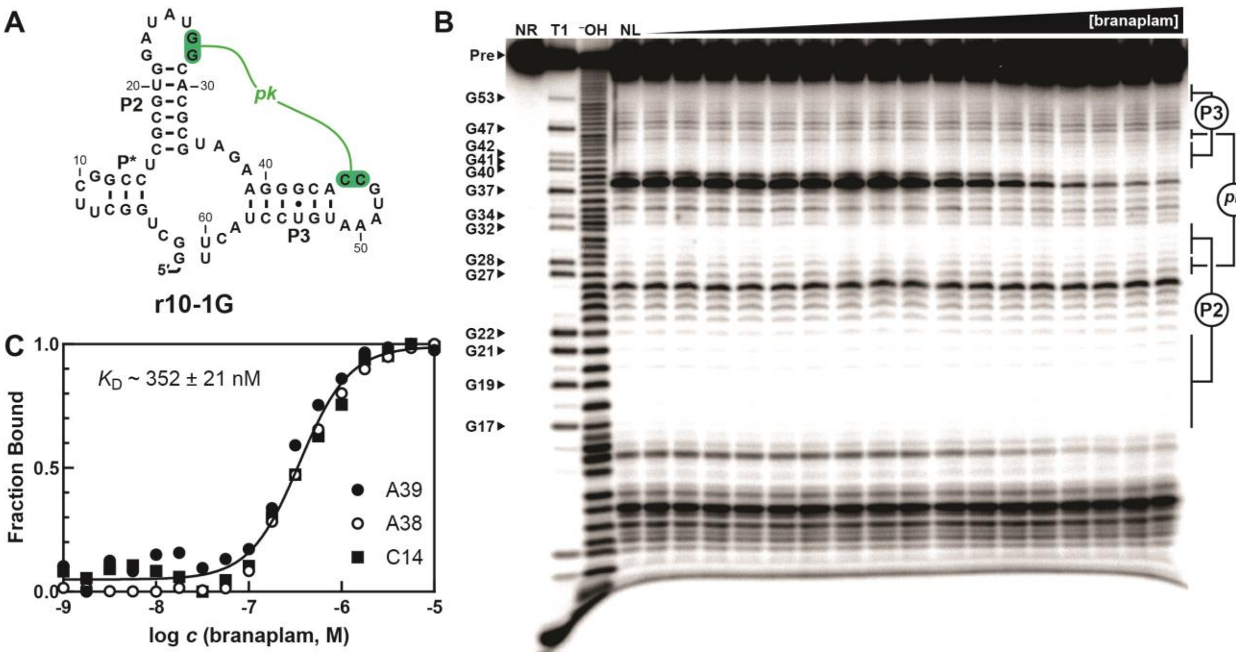

Figure S10. In-line probing of r10-1G with branaplam

A. Sequence and secondary structure model of r10-1G RNA. **B.** Autoradiogram of in-line probing gel with 5′-^{32}P-labeled r10-1G RNA incubated with increasing branaplam concentrations ranging from 10^{-9} to 10^{-5} M at quarter-log intervals. **C.** Plot of the logarithm of branaplam concentration vs. the fraction of RNA bound. The K_D for this interaction is 352 ± 21 nM.

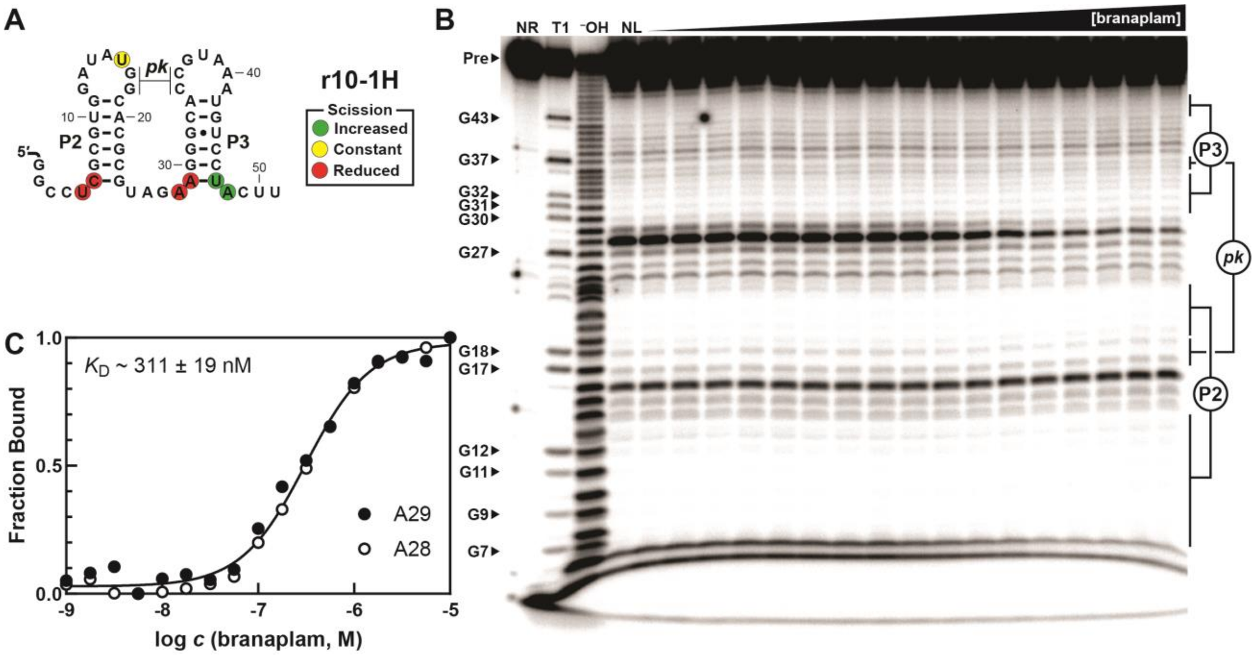

Figure S11. In-line probing of r10-1H with branaplam

A. Sequence and secondary structure model of r10-1H RNA. **B.** Autoradiogram of in-line probing gel with 5′-^{32}P-labeled r10-1H RNA incubated with increasing branaplam concentrations ranging from 10^{-9} to 10^{-5} M at quarter-log intervals. **C.** Plot of the logarithm of branaplam concentration vs. the fraction of RNA bound. The K_D for this interaction is 311 ± 19 nM.

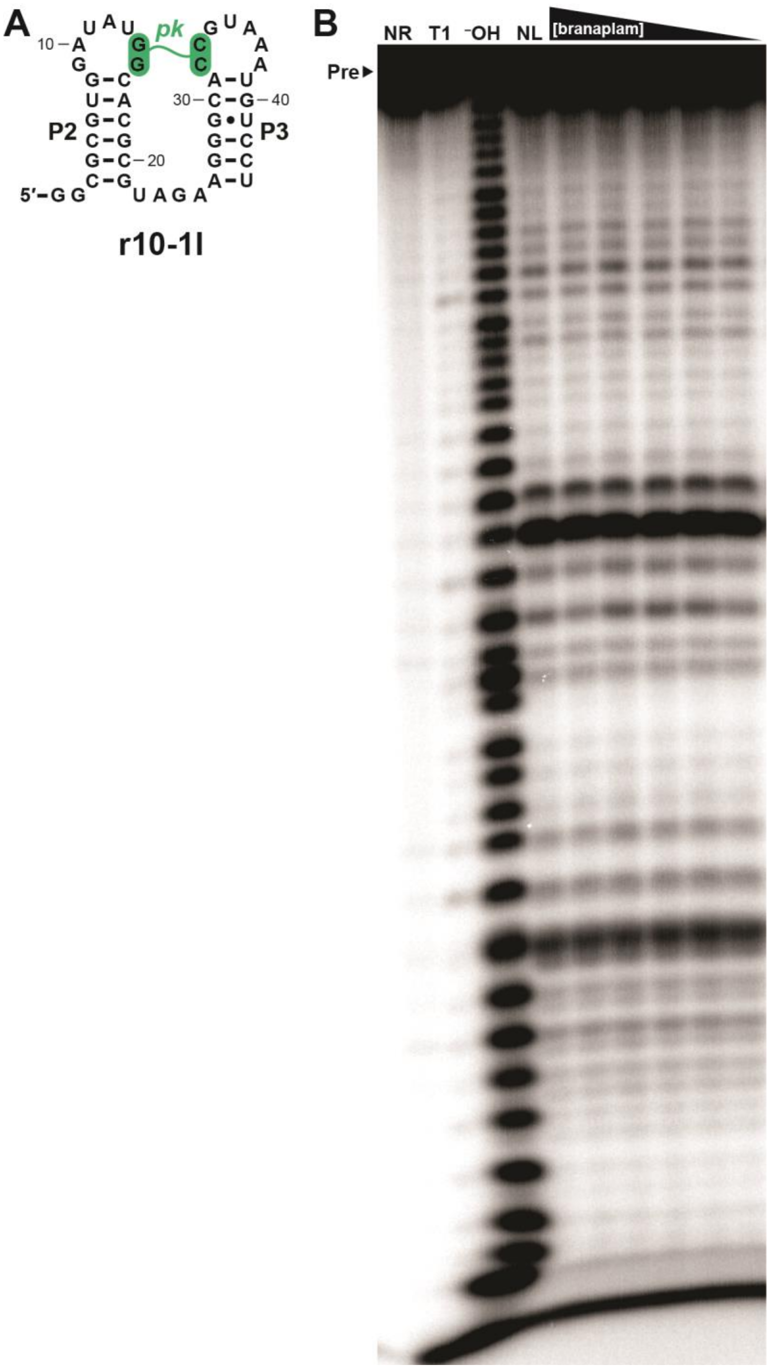

Figure S12. In-line probing of r10-1I with branaplam

A. Sequence and secondary structure model of r10-1I RNA. **B.** Autoradiogram of in-line probing gel with 5′-^{32}P-labeled r10-1I RNA incubated with decreasing branaplam concentrations ranging from 10^{-5} to 10^{-7} M at half-log intervals.

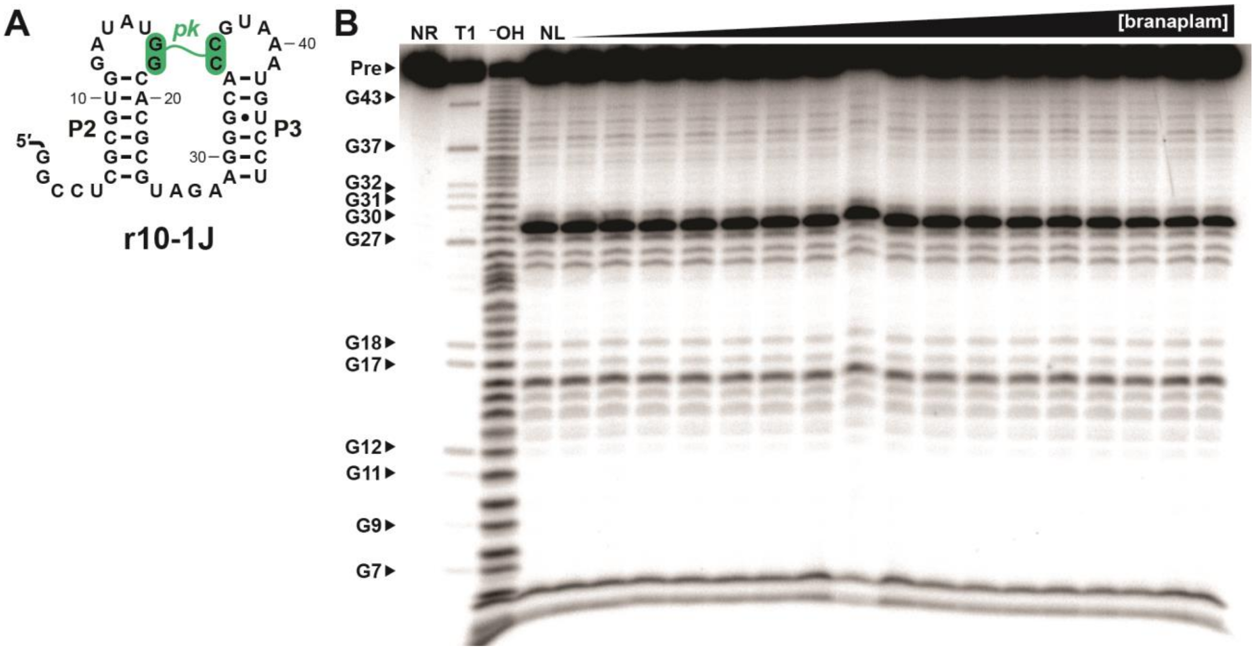

Figure S13. In-line probing of r10-1J with branaplam

A. Sequence and secondary structure model of r10-1J RNA. **B.** Autoradiogram of in-line probing gel with 5′-^{32}P-labeled r10-1J RNA incubated with decreasing branaplam concentrations ranging from 10^{-5} to 10^{-9} M at quarter-log intervals.

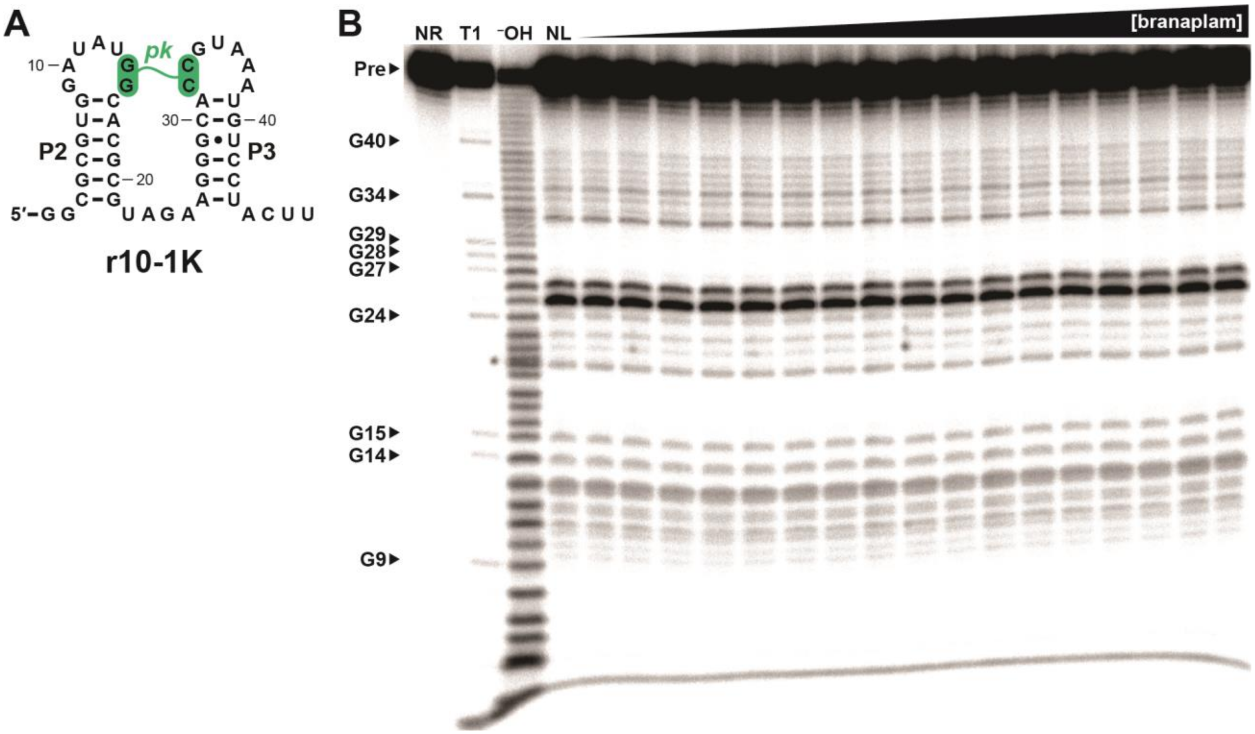

Figure S14. In-line probing of r10-1K with branaplam

A. Sequence and secondary structure model of r10-1K RNA. **B.** Autoradiogram of in-line probing gel with 5′-^{32}P-labeled r10-1K RNA incubated with decreasing branaplam concentrations ranging from 10^{-5} to 10^{-9} M at quarter-log intervals.

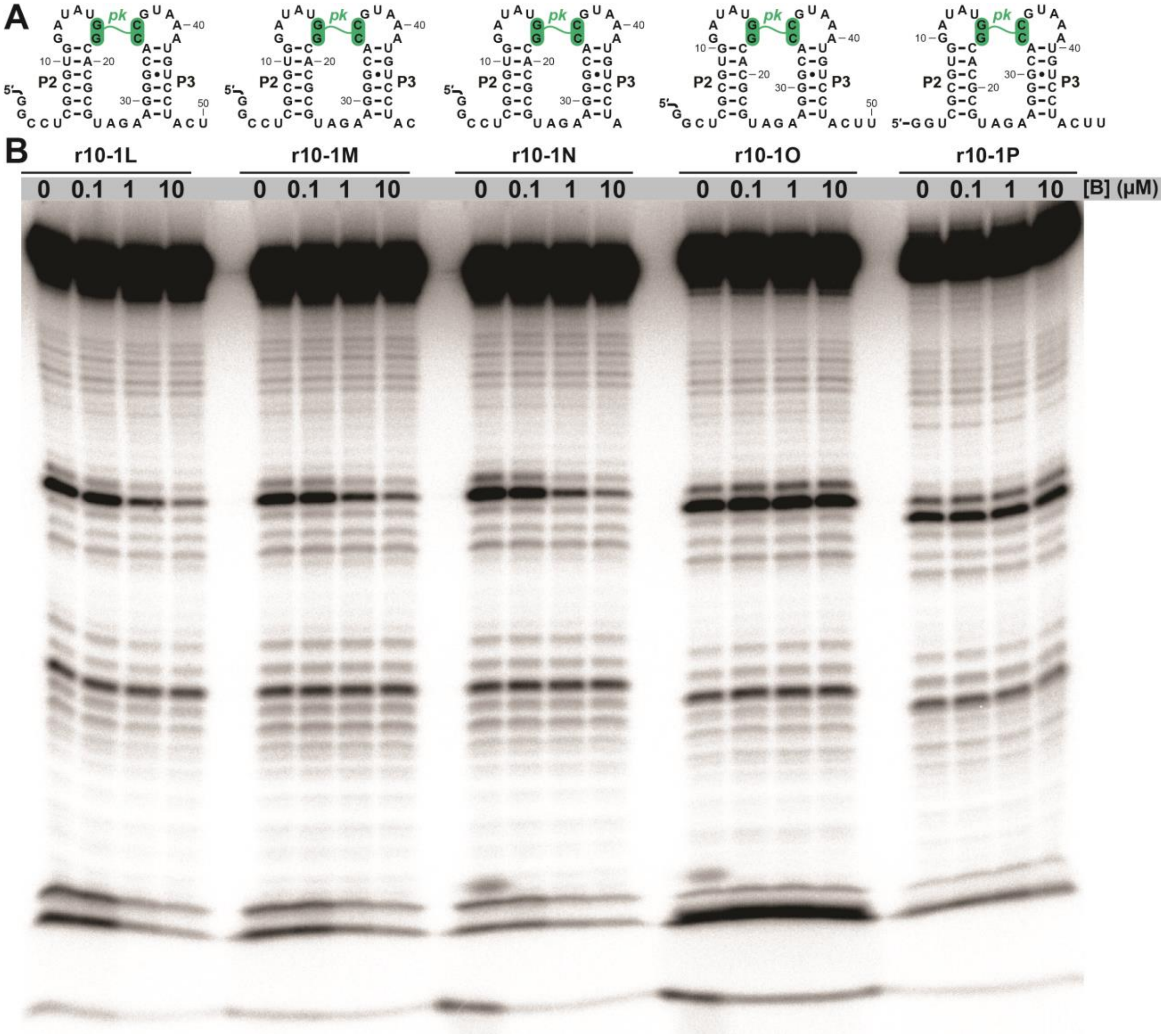

Figure S15. In-line probing of r10-1L through r10-1P with branaplam

A. Sequences and secondary structure models of RNAs r10-1L through r10-1P. **B.** Autoradiogram of in-line probing gel with the indicated 5′-^{32}P-labeled RNAs incubated with increasing branaplam concentrations ranging from 0.1 to 10 μM.

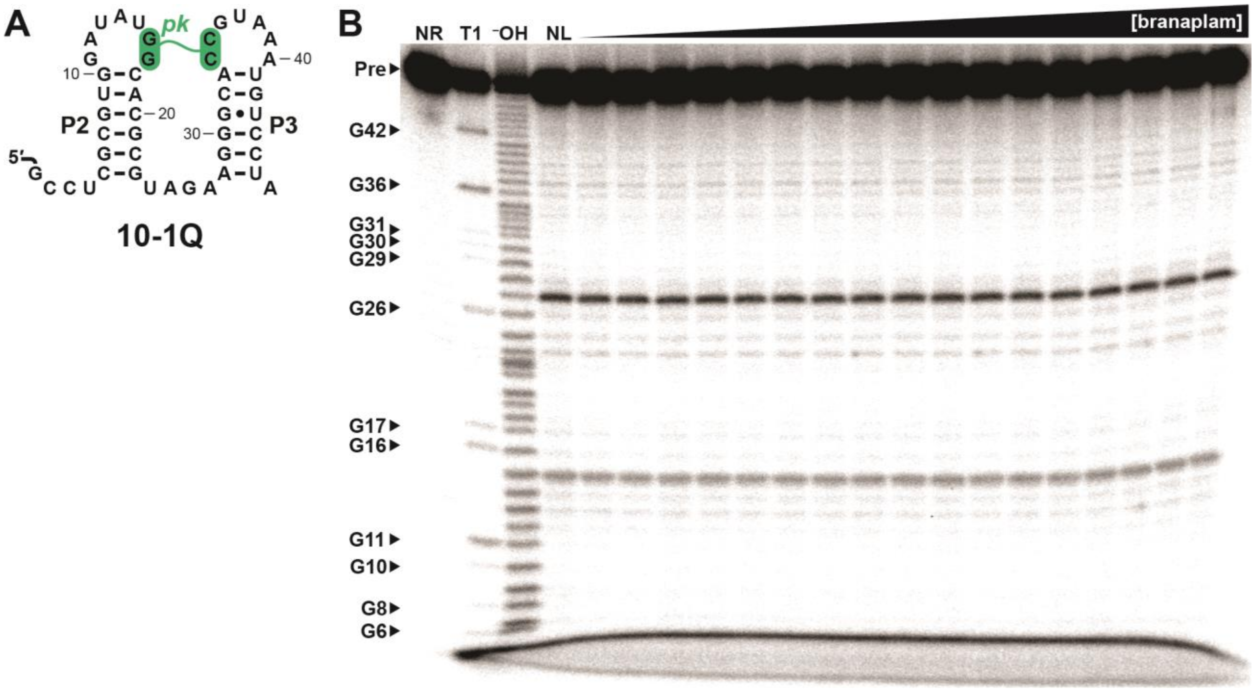

Figure S16. In-line probing of r10-1Q with branaplam

A. Sequence and secondary structure model of r10-1Q RNA. **B.** Autoradiogram of in-line probing gel with the indicated 5′-^{32}P-labeled RNAs incubated with increasing branaplam concentrations ranging from 10^{-5} to 10^{-9} M at quarter-log intervals.

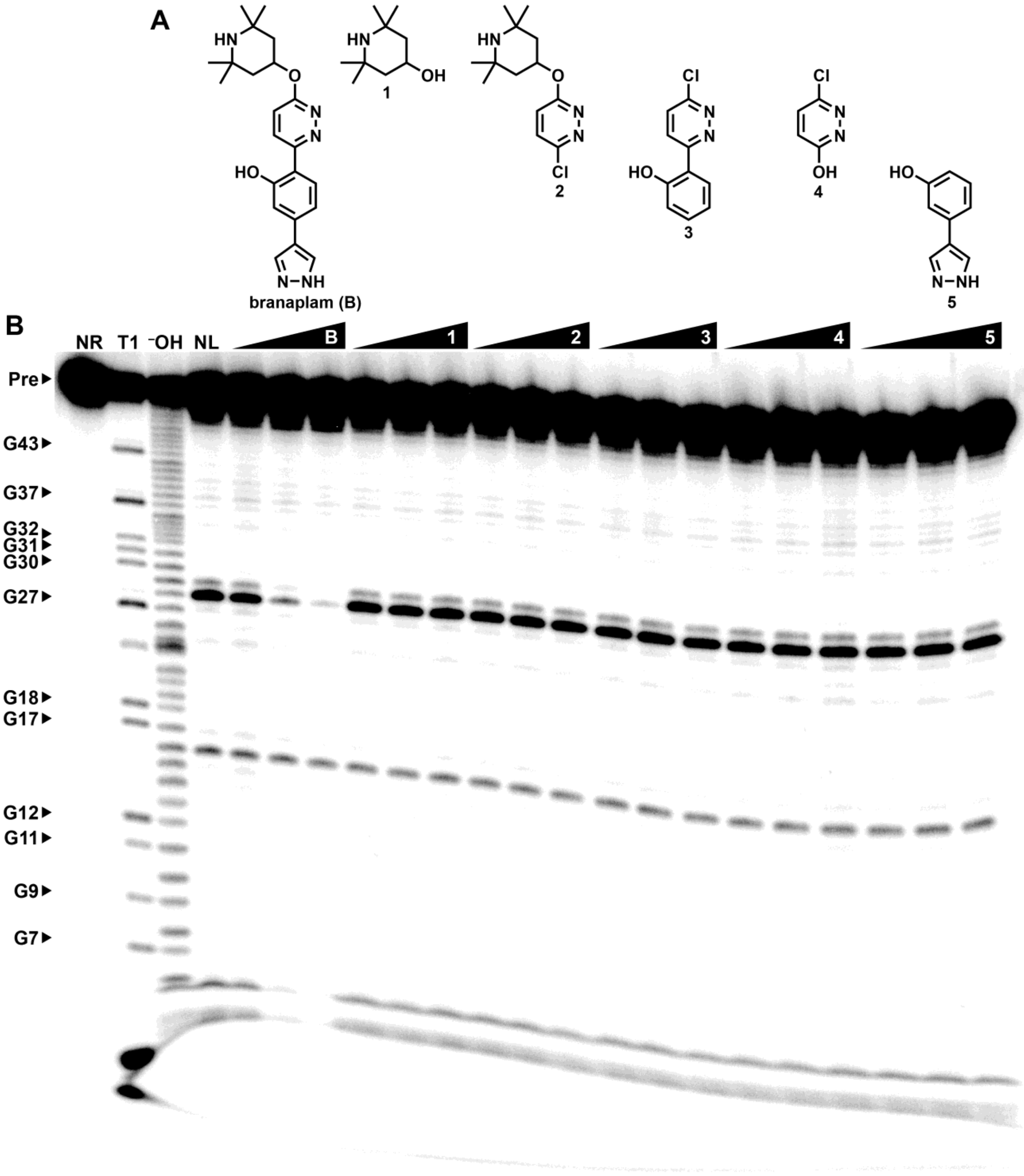

Figure S17. In-line probing of r10-1H with branaplam derivatives

A. Chemical structures of branaplam and derivatives **1** through **5** tested in this structure-activity relationship experiment. See **Methods** section for chemical names. **B.** In-line probing with 5′-^{32}P-labeled r10-1H RNA (see **Figure S11** for secondary structure model) incubated with increasing concentrations of the indicated compound ranging from 0.1 to 10 μM.

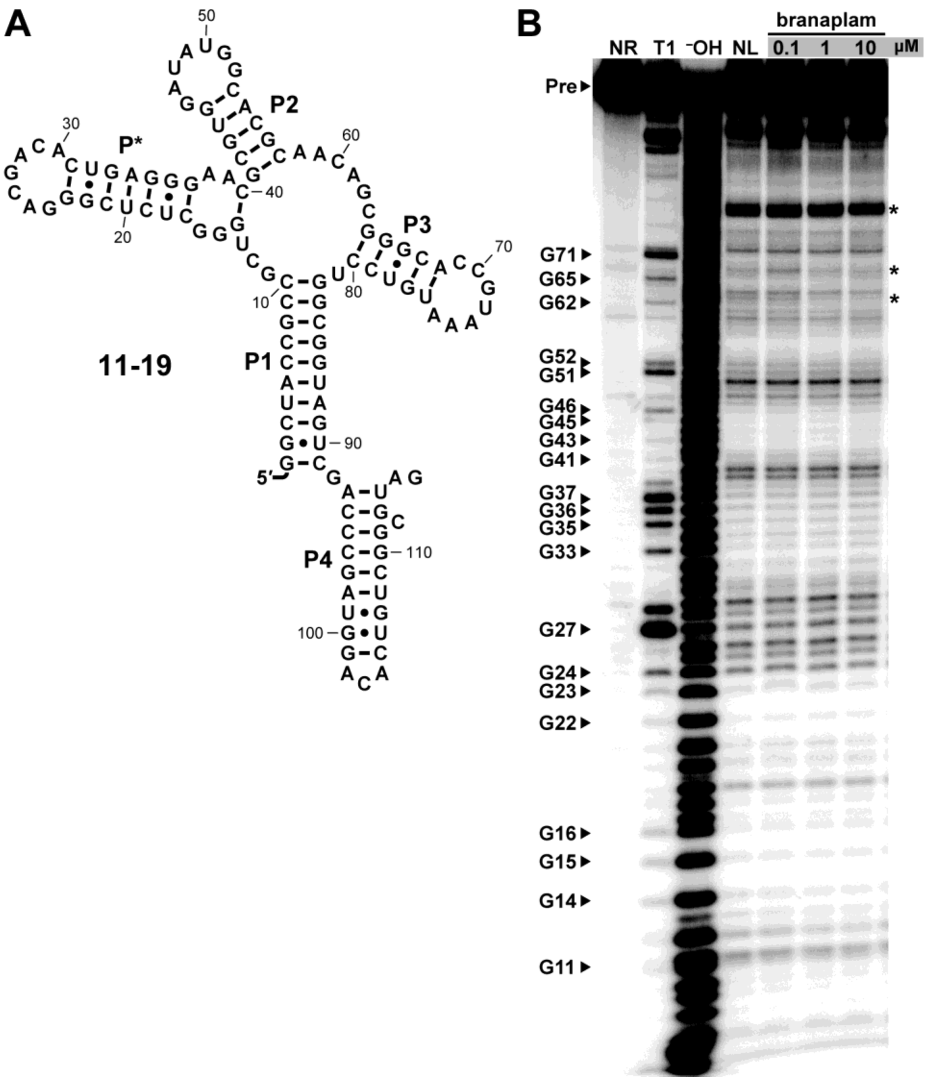

Figure S18. In-line probing of 11-19 with branaplam

A. Sequence and secondary structure model of 11-19 RNA. **B.** Autoradiogram of in-line probing gel with the indicated 5′-^{32}P-labeled 11-19 RNA incubated with increasing branaplam concentrations ranging from 0.1 to 10 µM. Modulating sites are indicated by asterisks (*).

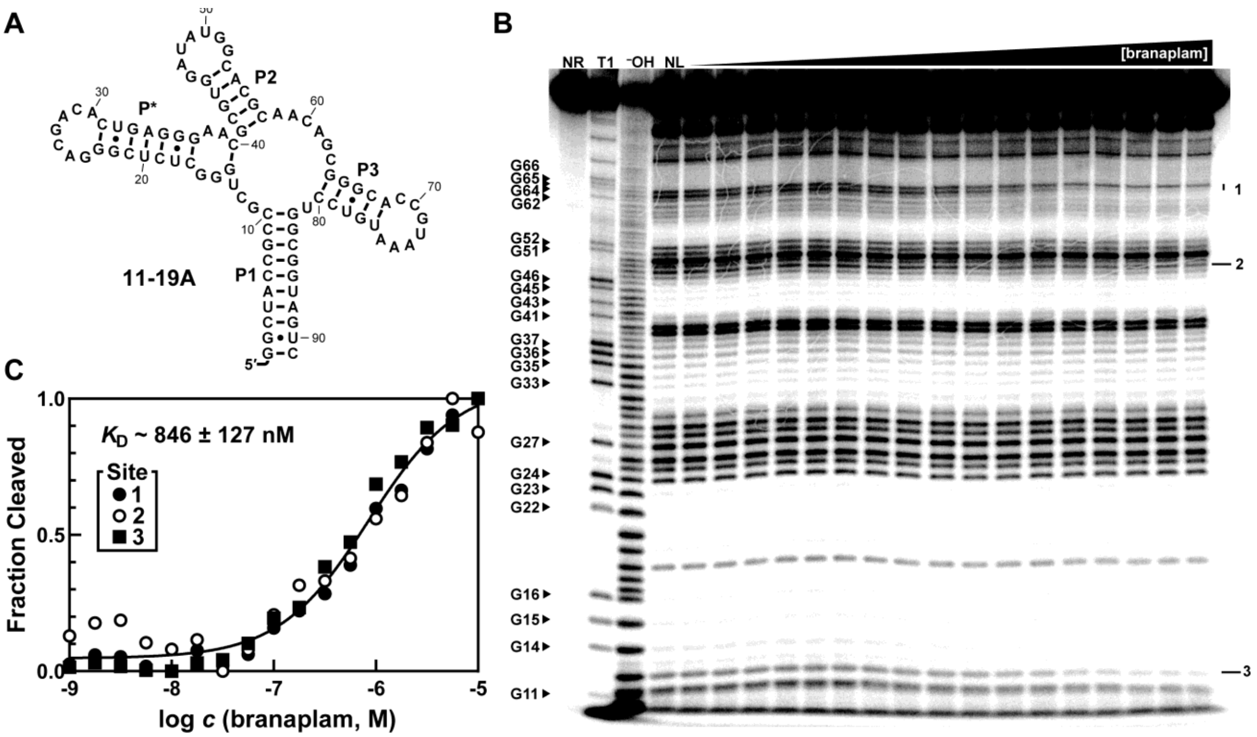

Figure S19. In-line probing of 11-19A with branaplam

A. Sequence and secondary structure model of truncated 11-19A RNA. **B.** Autoradiogram of in-line probing gel with 5′-^{32}P-labeled 11-19A RNA incubated with increasing branaplam concentrations ranging from 10^{-9} to 10^{-5} M at quarter-log intervals. **C.** Plot of the logarithm of branaplam concentration vs. the fraction of RNA bound. The K_D for this interaction is 846 ± 127 nM.

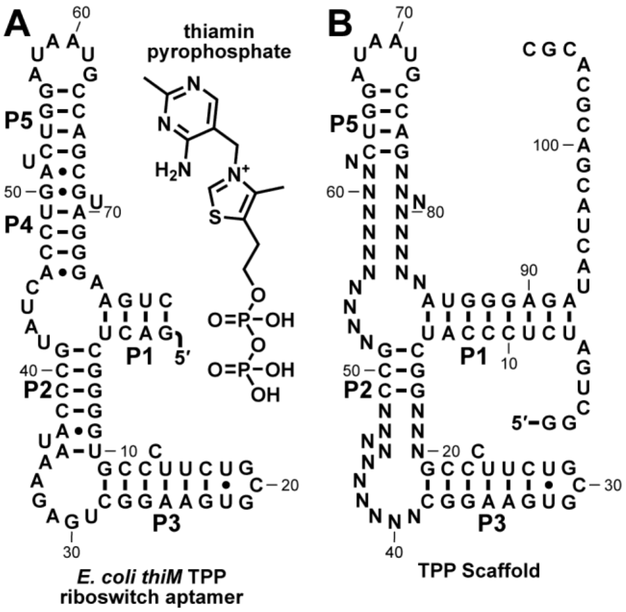

Figure S20. A scaffold inspired by a natural TPP riboswitch aptamer

A. Sequence and Sequence and secondary structure model of the TPP riboswitch aptamer located upstream of the *thiM* gene in *Escherichia coli*.[6,7] The natural ligand thiamin pyrophosphate is shown to the right. **B.** The TPP scaffold contains portions of P1, P2, P3, and P5 from the *E. coli thiM* riboswitch aptamer interspersed with regions of random sequence.

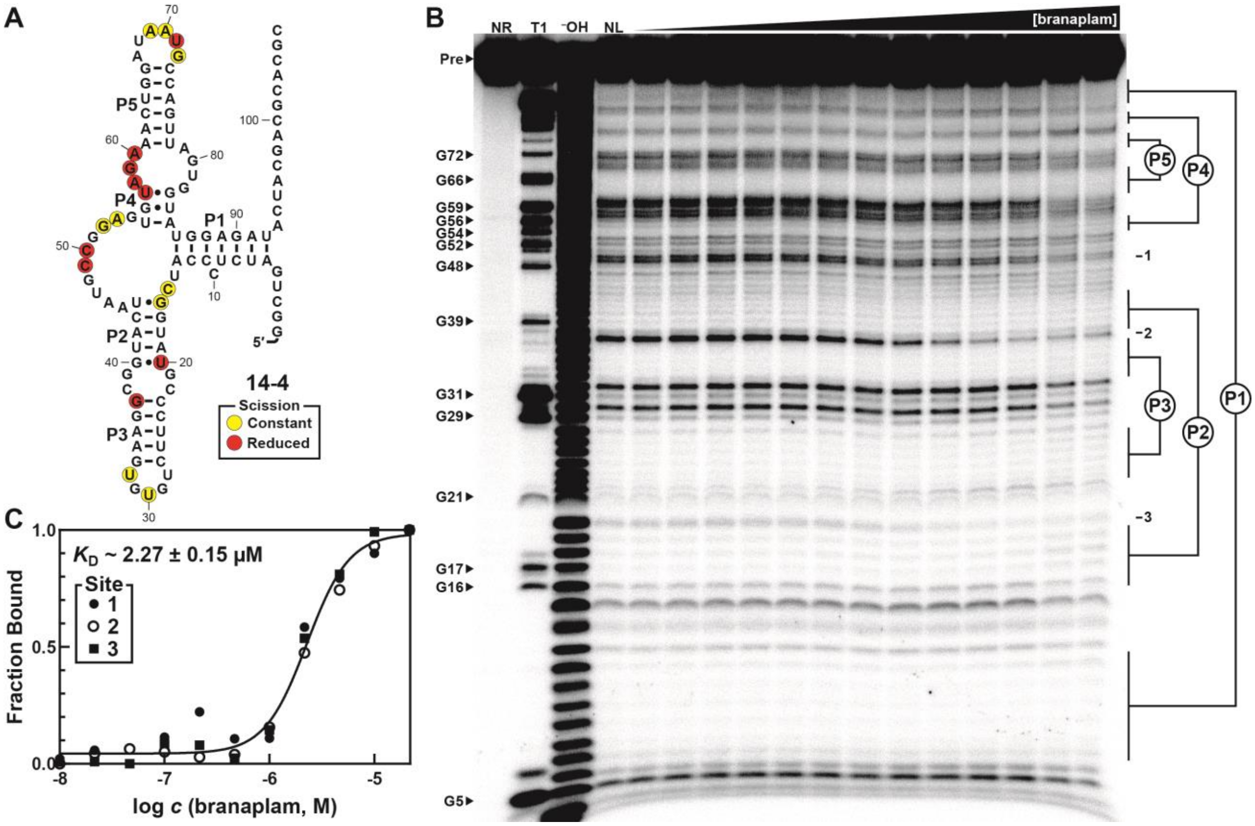

Figure S21. In-line probing of t14-4 with branaplam

A. Sequence and secondary structure model of t14-4 RNA. **B.** Autoradiogram of in-line probing gel with 5′-^{32}P-labeled t14-4 RNA incubated with increasing branaplam concentrations ranging from 10^{-8} to $10^{-4.67}$ M at third-log intervals. **C.** Plot of the logarithm of branaplam concentration vs. the fraction of RNA bound. The K_D for this interaction is 2.27 ± 0.15 μM.

REFERENCES

(1) Mohsen, M. G.; Breaker, R. R. In Vitro Selection and in Vivo Testing of Riboswitch-Inspired Aptamers. *Bio-protocol* **2023**, *13*.

(2) Regulski, E. E.; Breaker, R. R. In-Line Probing Analysis of Riboswitches. *Methods Mol. Biol.* **2008**, *419*, 53–67.

(3) Mohsen, M. G.; Midy, M. K.; Balaji, A.; Breaker, R. R. Exploiting Natural Riboswitches for Aptamer Engineering and Validation. *Nucleic Acids Res.* **2023**, *51*, 966–981.

(4) Yao, Z.; Weinberg, Z.; Ruzzo, W. L. CMfinder - A Covariance Model Based RNA Motif Finding Algorithm. *Bioinformatics* **2006**, *22*, 445–452.

(5) Weinberg, Z.; Breaker, R. R. R2R - Software to Speed the Depiction of Aesthetic Consensus RNA Secondary Structures. *BMC Bioinformatics* **2011**, *12*, 1–9.

(6) Winkler, W.; Nahvi, A.; Breaker, R. R. Thiamine Derivatives Bind Messenger RNAs Directly to Regulate Bacterial Gene Expression. *Nature* **2002**, *419*, 952–956.

(7) Serganov, A.; Polonskaia, A.; Phan, A. T.; Breaker, R. R.; Patel, D. J. Structural Basis for Gene Regulation by a Thiamine Pyrophosphate-Sensing Riboswitch. *Nature* **2006**, *441*, 1167–1171.
